# Adipocyte-specific deletion of sine oculis homeobox homolog 1 inhibits lipolysis and reduces skin fibrosis

**DOI:** 10.1172/jci.insight.181427

**Published:** 2026-02-19

**Authors:** Nancy Wareing, Tingting W. Mills, Scott Collum, Minghua Wu, Lucy Revercomb, René A. Girard, Hui Liu, Alexes Daquinag, Mikhail Kolonin, Marka Lyons, Brian Skaug, Weizhen Bi, Meer A. Ali, Haniyeh Koochak, Anthony R. Flores, Yuntao Yang, W. Jim Zheng, William R. Swindell, Shervin Assassi, Harry Karmouty-Quintana

**Affiliations:** 1 Department of Biochemistry and Molecular Biology, McGovern Medical School, University of Texas Health Science Center at Houston (UTHealth Houston), Texas, USA.; 2 Division of Rheumatology, Department of Internal Medicine, McGovern Medical School, UTHealth Houston, Houston, Texas, USA.; 3 Rice University, Houston, Texas, USA.; 4 Center for Metabolic and Degenerative Diseases, Institute of Molecular Medicine, UTHealth Houston, Houston, Texas, USA.; 5 D Bradley McWilliams School of Biomedical Informatics, Department of Internal Medicine, and; 6 Department of Pediatrics, McGovern Medical School, UTHealth Houston, Houston, Texas, USA.; 7 Department of Internal Medicine, University of Texas Southwestern Medical Center, Dallas, Texas, USA.; 8 Division of Pulmonary, Critical Care, and Sleep Medicine, Department of Internal Medicine, McGovern Medical School, UTHealth Houston, Houston, Texas, USA.

**Keywords:** Cell biology, Dermatology, Adipose tissue, Fibrosis, Skin

## Abstract

Dermal fibrosis is a cardinal feature of systemic sclerosis (SSc) for which there are limited effective disease-modifying therapies. SSc is characterized by dermal fibrosis accompanied by loss of dermal white adipose tissue (DWAT), yet the mechanisms linking adipocyte depletion to fibroblast activation remain unclear. Here we identify the transcription factor SIX1 as a central regulator coupling adipogenic repression with profibrotic signaling. SIX1 expression was increased in skin biopsies from 2 independent SSc cohorts and localized to fibroblast and perivascular stromal cells. In mice, ubiquitous or adipocyte-specific deletion of *Six1* preserved DWAT, reduced collagen accumulation, and selectively decreased profibrotic mediators. In cultured fibroblasts, CRISPR/Cas9-mediated *Six1* loss enhanced adipogenic markers while reducing profibrotic mediators and directly suppressed PAI-1 (*SERPINE1*) promoter activity. Together, these data position SIX1 as a transcriptional switch that promotes adipocyte reprogramming and fibrotic progression, and they highlight SIX1 inhibition as a potential therapeutic strategy to preserve adipocyte identity and limit dermal fibrosis.

## Abstract 2

<strong>SIX1 is elevated in systemic sclerosis skin and linked to adipose-associated biology. Deleting SIX1 in adipocytes reduces fibrosis, making it a potential therapeutic target.</strong>

## Introduction

Systemic sclerosis (SSc; or scleroderma) is a rare and heterogeneous autoimmune connective tissue disorder (1, 2). The multiorgan dysfunction has been characterized as a triad of immune dysregulation, vasculopathy, and excessive extracellular matrix (ECM) deposition by myofibroblasts, leading to skin and internal organ fibrosis (2–4). Skin thickening and tightening is responsible for considerable morbidity in this debilitating disease (5, 6). The extent of skin fibrosis defines the 2 subclasses of SSc: limited cutaneous (lcSSc) and diffuse cutaneous SSc (dcSSc) (3). There are currently no FDA-approved treatments for skin involvement in SSc. More extensive skin involvement at the time of diagnosis is associated with higher levels of disability, severity of pain, and decreased survival (7, 8). This underscores the urgent need to identify novel targeted therapies for the treatment of SSc (9).

Thus, understanding the early pathogenesis of disease represents a critical step toward novel therapeutic approaches (9). The mechanisms that lead to organ fibrosis in SSc are not fully understood; however, similar mediators have been identified to play a role in the lung and skin. For example, increased adenosine, hyaluronan, and IL-6 have been implicated in the pathophysiology of both lung and skin fibrosis (10–14). However, these mechanisms have largely focused on fibroblast and epithelial biology, with limited studies in adipocytes. This is important as an early hallmark of SSc is skin-associated adipose tissue atrophy and replacement by ECM, leading to dermal thickening (15, 16). Clinically, this may directly contribute to rigidity and tethering observed in early lesional SSc skin (15, 16). Dermal white adipocyte tissue (DWAT) adipocytes display highly distinct features compared with other white adipocytes, including significant plasticity (17–19). They can cycle through dedifferentiation and redifferentiation as part of a physiologic response to hair cycling, aging, and energy demands (17, 18, 20). Under certain conditions, dedifferentiated preadipocytes escape the normal cycle of redifferentiation and transdifferentiate into ECM-producing myofibroblasts. This process is referred to as the adipocyte-to-myofibroblast transition (AMT) (21, 22). AMT has been well studied in wound healing (21, 23), and recent evidence suggests it may also contribute to pathological skin fibrosis (22, 24). Mature adipocytes in the skin produce PDGF ligands and BMPs, both of which are implicated in wound healing and fibrosis (25). In addition, the adipose secretome has also been identified to play a role in the pathophysiology of SSc (26). Lipid-filled adipocytes cross-talk with other cell types in the stroma-vascular fraction of adipose tissue. The interaction between the dermal fibroblasts and adipocytes has also been appreciated as a contributor to irregular inflammation and aberrant wound healing through proinflammatory signaling between cell types (27) and adipocyte-driven regulation of fibroblast ECM production (28). A robust physiologic axis also exists between adipocytes and endothelial cells, by which cell signaling can be bidirectionally regulated (29). However, mechanisms by which adipocytes contribute to the pathogenesis of skin fibrosis in SSc remain poorly understood.

Our group and others identified sine oculis homeobox homolog 1 (SIX1) as a novel mediator in lung fibrosis, promoting the release of profibrotic mediators by alveolar epithelial cells (30–32). Furthermore, SIX1 has also been implicated in asthmatic lung fibrosis (31, 32) and in liver fibrosis defined by excessive myofibroblast activation and ECM deposition (33). Lung fibrosis is an important complication of SSc that is typically observed following onset of skin fibrosis, and while epithelial and fibroblast-based mechanisms are most highly studied, the contribution of the adipocyte to the fibrotic process is not fully known (34). However, whether SIX1 plays a role in skin fibrosis in SSc is not known; in particular, how adipocyte SIX1 regulates dermal fibrosis remains underinvestigated. SIX1 is a member of an evolutionarily conserved family of developmental transcription factors (35). SIX1 plays a critical role in regulating the expression of genes that control precursor cell survival and proliferation during embryogenesis. In healthy adults, SIX1 is negligibly expressed in most tissues (36). Perhaps the most well-studied role of SIX1 in adulthood is in the context of cancer, where it is a critical regulator of transdifferentiation of precancerous cells into mesenchymal cells with metastatic features (37–41). Although *SIX1* transcript expression has been identified in healthy s.c. adipose tissue (42), to date, its exact role in adipose tissue biology remains poorly investigated. Brunmeir et al. (43) were the first to identify the direct transcription regulation by SIX1 and the interaction between SIX1 and major regulators of adipogenesis, in mature fat cells. Recently, it was demonstrated that in vivo SIX1 overexpression in mouse hepatocytes exacerbates diet-induced liver inflammation, metabolic disruption, and hepatic steatosis as well as activates liver-specific receptors to induce de novo lipogenesis (44). This work is founded upon the fundamental and newly developing understanding of the roles of SIX1, particularly in the realm of lipolysis and the release of profibrotic factors that regulate dermal fibrosis. We hypothesize that SIX1 contributes to dermal lipoatrophy and skin fibrosis in SSc. Surprisingly, our data suggest that SIX1 is not involved in modulating adipocyte phenotype, but instead, it regulates the release of the profibrotic mediator, PAI-1 that promotes dermal fibrosis.

## Results

### Increased SIX1 levels correlate to skin fibrosis in patients with SSc.

**Figure 1:**
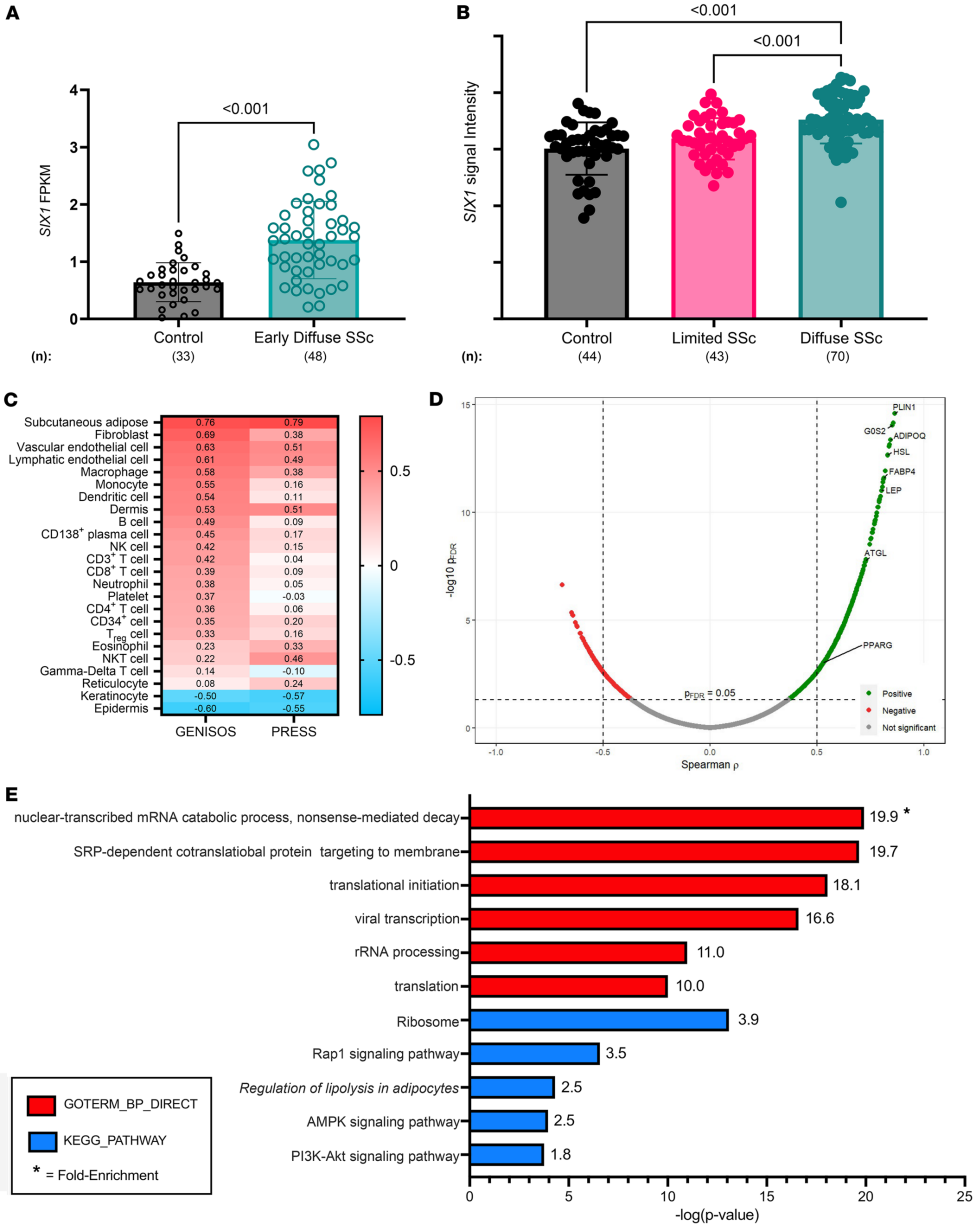
*SIX1* is elevated in SSc skin and correlates with adipose-related genes and pathways. (**A**) *SIX1* expression in PRESS SSc skin samples and controls. FPKM, fragments per kilobase million based on a FDR cutoff of 0.05 and fold change cutoff of > 1.5 or < 0.6 (**B**) *SIX1* expression in baseline SSc skin samples and controls in the GENISOS cohort based on Student’s *t* test. (**C**) Heatmap showing correlation (*r*) between skin *SIX1* expression and cell type signature scores on Spearman’s rank order correlation. Bolded pathways indicate correlations with *P* < 0.05 in both cohorts. (**D**) Volcano plot showing individual gene-*SIX1* correlations in PRESS cohort SSc skin samples. PLIN1, perilipin 1; G0S2, G0/G2 switch gene 2; ADIPOQ, adiponectin; HSL, hormone sensitive lipase; FABP4, fatty acid binding protein 4; LEP, leptin; ATGL, adipose triglyceride lipase; PPARG, peroxisome proliferator activated receptor γ based on a Spearman’s rank coefficient analysis. (**E**) KEGG pathway annotation of all DEGs in SSc skin of PRESS cohort participants using GoStats. Significancy levels refer to a Bonferroni cut-off of 0.5 analysis (**A**) or a the R Bioconductor package edgeR6 analysis for to identify differentially expressed transcripts between patients with SSc and healthy controls with a FDR cutoff of 0.05 and fold change cutoff of > 1.5 or < 0.67 for **B**.

Previously, our group identified increased SIX1 in lung fibrosis (30), and *SIX1* expression has been identified to be present in s.c. adipocytes, particularly in those exhibiting aberrant function (42). Therefore, we aimed to determine whether *SIX1* was elevated in skin samples from patients with SSc. To do this, we first determined expression of *SIX1* from 2 distinct cohorts: The Genetics versus ENvironment In Scleroderma Outcome Study (GENISOS) cohort, which includes patients with lcSSc and dcSSc at different stages of disease, and the Prospective Registry for Early Systemic Sclerosis (PRESS) cohort, enriched for patients with early-stage dcSSc. *SIX1* transcript levels were elevated in SSc skin in limited SSc, diffuse SSc, and in early diffuse SSc compared with control skin in both independent cohorts (Figure 1). In the PRESS cohort, which is enriched for patients with early dcSSc, RNA-seq revealed increased *SIX1* signals (Figure 1A). In the GENISOS cohort, which encompasses dcSSc and lcSSc at different stages of disease, *SIX1* signal intensity, denoting expression levels, was higher in patients with dcSSc compared with lcSSc (Figure 1B). To determine whether *SIX1* is associated with the genomic landscape of a particular cell type, we correlated *SIX1* expression levels with cell type–specific signature scores previously utilized by our group (45, 46). The bioinformatic analysis identified genes that are expressed at comparatively higher levels in a specific cell type and created a signature score for each cell type being evaluated. The s.c. adipose signature was the most highly correlated with *SIX1* expression in both the GENISOS (r = 0.76) and PRESS cohorts (r = 0.79). This points to *SIX1* is an important mediator that is elevated in adipose tissue in SSc (Figure 1C). Higher expression of genes specific to fibroblasts, vascular and lymphatic endothelial cells, significantly correlated with higher expression of *SIX1*, suggesting that *SIX1* may regulate mesodermal derived cells during fibrosis but not the ectodermally derived epithelium. Individual gene correlation analysis revealed that genes associated with adipocyte biology were enriched among those genes most highly correlated with *SIX1*, specifically in early dcSSc skin (Figure 1D and Supplemental Table 3; supplemental material available online with this article; https://doi.org/10.1172/jci.insight.181427DS1). These included genes encoding proteins required for adipocyte differentiation and triglyceride metabolism, including *ADIPOQ* and *PPARG* (47–49). Functional annotation of all differentially expressed genes (DEGs) in the skin of patients with dcSSc in the PRESS cohort compared with healthy controls showed significant enrichment for “regulation of lipolysis in adipocytes” in addition to “ribosome” and “AMPK signaling pathway” (Figure 1E).

### Increased adipocyte SIX1 levels correlate with loss of dermal white adipose tissue (DWAT) in SSc.

**Figure 2:**
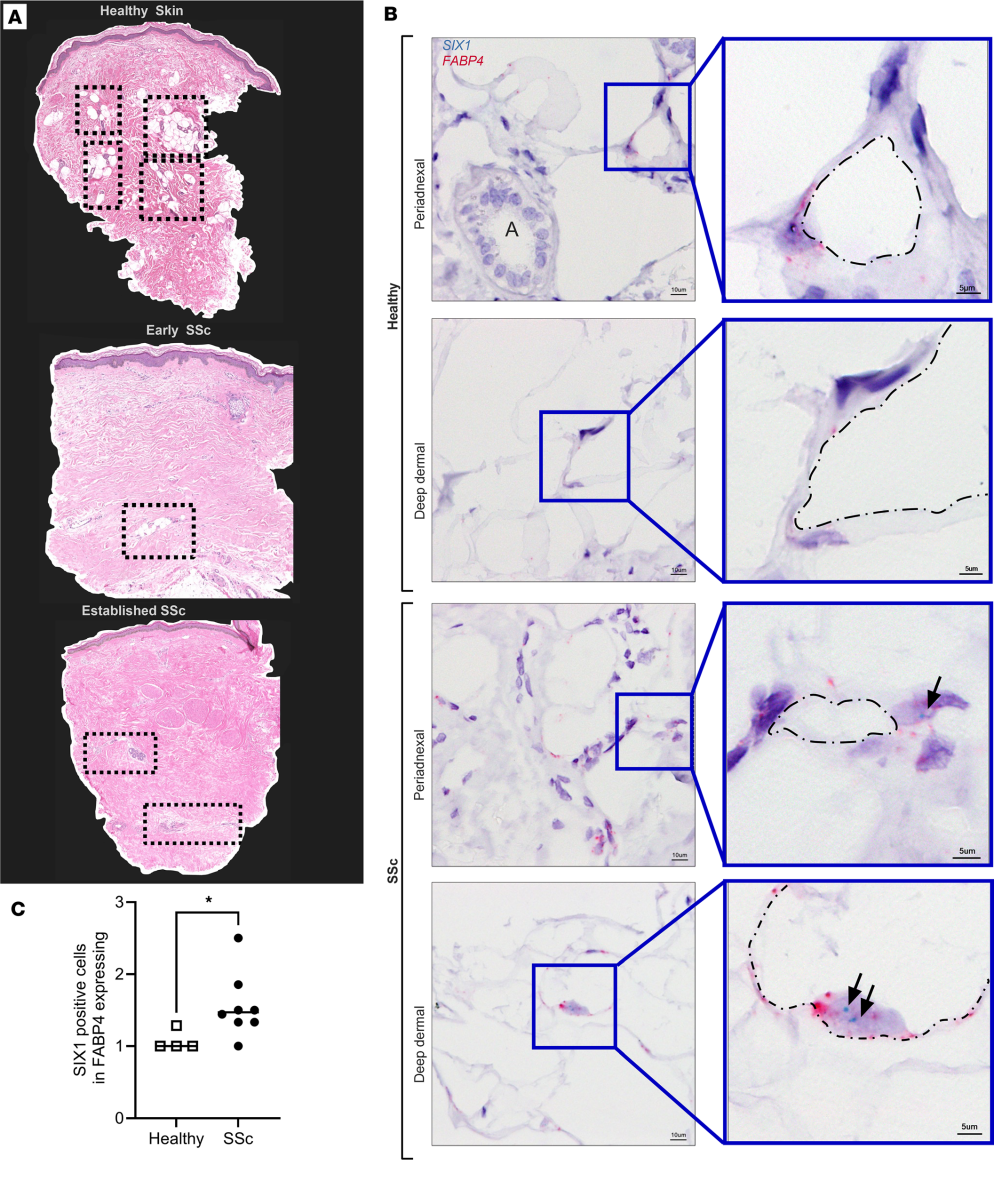
Dermal White Adipose Tissue (DWAT) atrophy and increased adipocyte SIX1 expression in SSc. (**A**) H&E staining of human skin biopsies from GENISOS cohort participants. Top: control skin. Middle: early SSc-representative image. Bottom: established representative SSc-skin sample. Clinical and demographic features of biopsies individuals are provided in Supplemental Table 4. Boxes contain dermal white adipose tissue. Dotted boxes denote DWAT areas. (**B**) Representative images of dual in situ hybridization for *SIX1* (teal) and *FABP4* (pink) in 3 SSc and 1 demographically matched control biopsies. Hematoxylin (purple) costain labels nuclei. “A” marks a dermal adnexal structure. Black arrows point to *SIX1* transcript signal. Dash dot outlines denote the interior periphery of adipocytes. (**C**) Representative morphometric quantification for dual positive FABP4/SIX1 cells in healthy versus SSc tissue. *P ≤ 0.05 using Mann-Whitney *U* comparisons between healthy (*n* = 4) versus SSc (*n* = 8).

Several studies have shown that the lipoatrophy and loss of DWAT is present in skin fibrosis in SSc (15) and even precedes the fibrotic matrix deposition (50). In addition, AMT (22, 24) and the adipose secretome (26) have been implicated in the pathophysiology of SSc. Thus, we next aimed to determine whether *SIX1* levels were increased in the diminishing DWAT layer in the skin. Herein, we assessed tissue samples from the GENISOS cohort that include SSc-affected skin samples with varying disease durations, compared with an age, sex, and ethnicity-matched control sample, demonstrating a progressive loss of DWAT areas as disease progresses (Figure 2A). This is important as DWAT levels are known to reduce as dermal fibrosis develops, thus the GENISOS cohort allows us to temporally assess loss of adipose tissue and SIX1 levels during progression of disease. Demographic and clinical features of these individuals are listed in Supplemental Table 4. Compared with the control biopsy, there was notably less DWAT in the skin of all dcSSc-affected individuals, with the patient with the most established form of disease presenting with no discernible DWAT areas (Figure 2A). Thus, having established a correlation between changes in adipocyte biology, stromal cells, and *SIX1* gene expression in SSc skin; we sought out to identify those cell types that produce *SIX1* in SSc skin with abnormal dermal fat.

We selected 8 patients with SSc who retained dermal fat, most of whom were within 3 years of developing diffuse disease. Additionally, skin biopsies from 4 healthy controls were included. To localize the *SIX1* gene in these samples, we employed single-molecule in situ hybridization. DWAT in human skin is localized around adnexal glands and hair follicles, and within and below the deep dermis (51). *SIX1* was detected in both periadnexal and deep dermal adipocytes. Mature adipocytes are identifiable by a single large lipid droplet surrounded by a thin ring of cytoplasm and a peripheral nucleus, which expresses Fatty Acid Binding Protein 4 (*FABP4*) (Figure 2B). Our morphometric quantification revealed increased expression of *SIX1* in SSc tissue (Figure 2C). We acknowledge that some fibroblasts also express *FABP4*; however, the unique morphology of adipocytes as described above makes them easily distinguishable from the small, spindle-shaped fibroblast with a dominant, central nucleus. Although FABP4^+^ cells are consistent with preadipocytes, it is plausible for a subset of cells to include macrophage- or vascular-associated populations. In addition, based on landmark scRNA-seq studies of SSc skin (52), SIX1 expression is increased in fibroblast and pericyte subtypes, with limited expression in keratinocytes and macrophages and reduced expression in endothelial cells. (Supplemental Table 5). It is important to note that no datasets were present for mature adipocytes. The clinical relevance of these findings was supported by positive correlations between the expression of *SIX1* in SSc skin and the extent and severity of SSc skin fibrosis. Spearman’s rank-order correlation analysis showed a positive correlation between whole skin *SIX1* expression and modified Rodnan skin score (mRSS) (*r* = 0.40, *P* < 0.001), and local skin score (*r* = 0.38, *P* < 0.001) near the site of the biopsy. To our knowledge, dermal fat *SIX1* has not previously been identified in situ, the significance of which is supported by clinical data linking *SIX1* to more severe and extensive SSc skin involvement. Together, these data from 2 SSc study cohorts provided a strong premise for investigating *SIX1* in SSc disease mechanisms.

### Adipose tissue loss is evident in a bleomycin model of dermal fibrosis.

We selected the murine s.c. bleomycin (bleo) model of skin fibrosis (53) as the preclinical model to study the effects of *SIX1* on fibrosis and lipoatrophy (11, 54). Unlike human skin, rodent skin DWAT is separated from the SWAT by a thin layer of skeletal muscle (the panniculus carnosus), allowing us to distinguish dermal adipocytes from s.c. adipocytes without the use of additional markers (55, 56). Furthermore, the fibrotic changes that occur over the 28 days of s.c. bleo treatment allow us to study the role and expression of SIX1 during the pathogenesis of dermal fibrosis (22, 50, 57).

**Figure 3:**
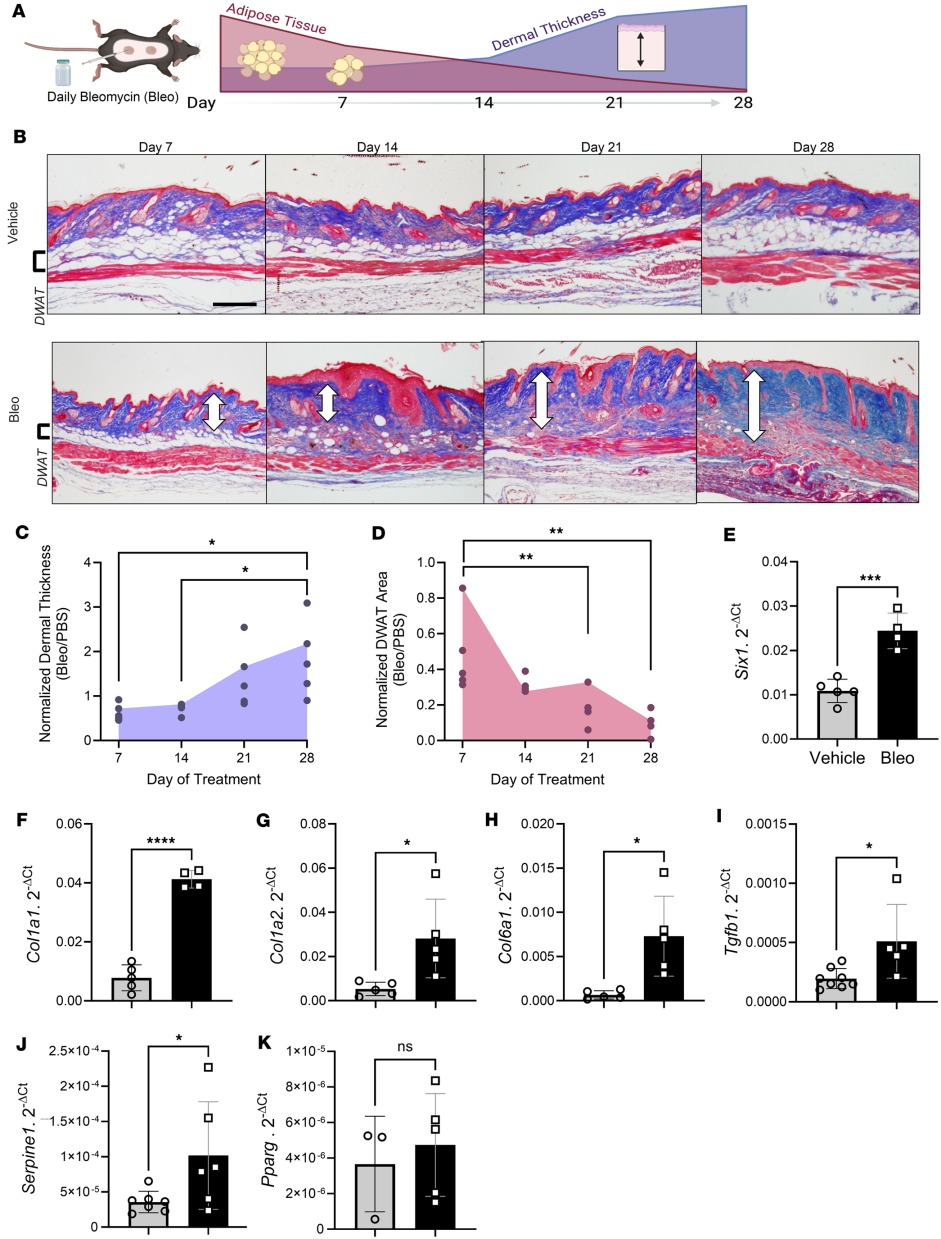
Dermal white adipose loss precedes fibrosis in the murine model of bleomycin induced skin fibrosis. (**A**) Schematic representation of s.c. bleo model of skin fibrosis in mice (created with BioRender). (**B**) Representative images of Masson’s trichrome staining of dorsal mouse skin after 7, 14, 21, and 28 days of s.c. vehicle or bleo treatment. White arrows indicate dermal thickness. Scale bar: 200 μm. (**C** and **D**) Dermal thickness and area of DWAT at 7, 14, 21, and 28 days reported in a dot plot, as mean ± SD of bleo-injected mice normalized to the average of all vehicle-injected mice at that time point. *n* = 4–5 for each time point. Transcript expression levels for sine oculis homeobox homolog 1 (*Six1*, **E**),collagen 1a1 (*Col1a1*, **F**), collagen 1a2 (*Col1a2*, **G**), collagen 6a1 (*Col6a1*, **H**), TGF-β1 (*Tgfb1*, **I**), serpin family E member 1 (*Serpine1*, **J**), and peroxisome proliferator activated receptor γ (*Pparg*, **K**) in 7-day skin samples by qPCR. Expression was normalized to 18s rRNA. DWAT, dermal white adipose tissue. *P ≤ 0.05, **P ≤ 0.01, ***P ≤ 0.001, and ****P ≤ 0.0001 refer to a simple linear regression for **C** and **D** and an unpaired *t* test for **E**–**K**. Each individual plot represents a biological *n*. *n* = 3–7.

**Figure 4:**
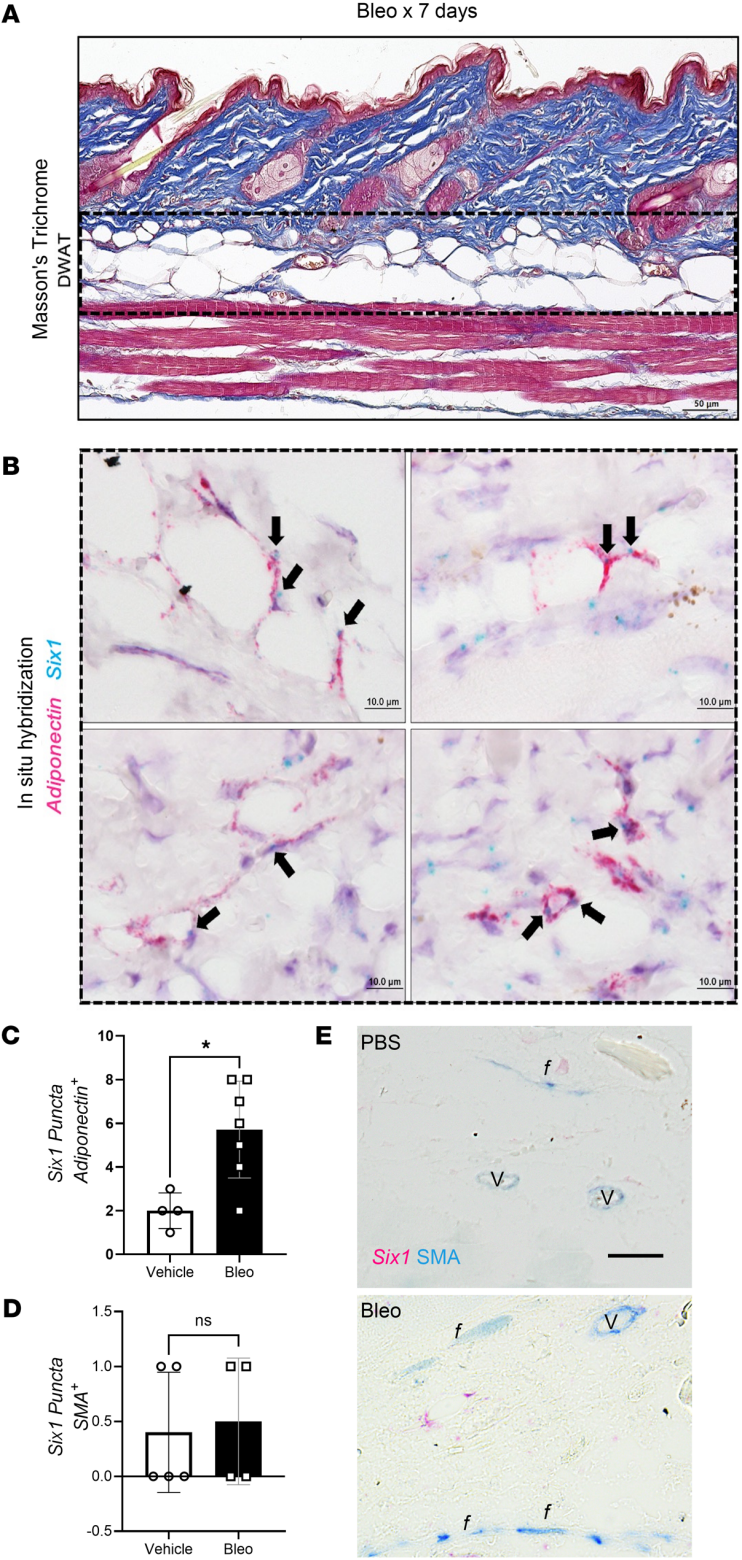
Adipocyte *Six1* expression precedes white adipose loss in s.c. bleomycin-treated mice. Representative images of mouse dorsal skin injected with s.c. bleo for 7 days (*n* = 6). (**A**) Masson’s trichrome staining. (**B**) Dual in situ hybridization for *Adiponectin* (pink) and *Six1* (teal). Arrows point to *Six1* signal. DWAT, dermal white adipose tissue. (**C**) Quantification of *Six1* puncta in Adiponectin expressing cells from vehicle (PBS, *n* = 4) or Bleo treated mice (*n* = 7). (**D**) Quantification of *Six1* puncta in smooth muscle actin (SMA) expressing cells from vehicle-treated (PBS, *n* = 5) or from Bleo-treated (*n* = 4) mice. (**E**) Dual IHC for SMA (blue signals) and RNAScope for *Six1*, magenta puncta fibroblasts *f* denote fibroblasts, and *v* refer to vessels. Scale bar: 50 μm (**A**), 10 μm (**B**), and 20 μm (**E**). *P ≤ 0.05 refers to an un unpaired *t* test for **C** and **D**.

We demonstrated that serial injections of s.c. bleo recapitulated lipodystrophy and dermal sclerosis mirroring SSc manifestations (Figure 3A) (22, 49, 56). Using Masson’s trichrome staining, we showed progressive bleo-induced atrophy of the DWAT, increased collagen deposition, and dermal thickening observable at weekly time points up to 28 days (Figure 3B). Attrition of DWAT was appreciable on histology as early as day 7 of bleo treatment, when dermal thickening was less pronounced. Quantification of dermal thickness and DWAT area confirmed these observations. When compared with day 7, dermal thickening was significant after 28 days of bleo (Figure 3C). This change lagged behind the significant decline in DWAT area (Figure 3D). Transcriptomic analysis revealed increased signals for *Six1* (Figure 3E), and despite minimal histological changes in the dermis after 7 days of bleo, prominent ECM genes collagen 1a1 (*Col1a1*), collagen 1a2 (*Col1a2*), and collagen 6a1 (*Col6a1*),TGF-β (*Tgfb1*), and of serine proteinase inhibitor E 1 (*Serpine1*) were upregulated (Figure 3, F–J). Intriguingly, we did not detect increased expression for peroxisome proliferator-activated receptor γ (*Pparg*) on day 7 of s.c. bleo (Figure 3K). These results support that a reduction in DWAT and upregulation of fibrotic genes precedes significant dermal expansion following s.c. bleo treatment. Next, we investigated whether adipocyte *Six1* expression, as observed in SSc skin, was recapitulated in the s.c. bleo model. Dual in situ hybridization probed for skin *Six1* and *Adipoq* transcript expression after 7 days of s.c. vehicle or bleo injections. *Adipoq* is highly and specifically expressed in lipid-laden adipocytes (48). Mirroring findings in human SSc skin whereby *SIX1* was elevated in early disease, *Six1* transcripts levels were upregulated in the dermal adipocytes after 7 days of s.c. bleo treatment (Figure 4, A–C). We also assessed *Six1* expression levels in fibroblasts using smooth muscle actin (SMA) as a readout; these studies did not reveal increased *Six1* in SMA^+^ cells. (Figure 4, D and E). Collectively, these data demonstrate that adipocyte loss is evident prior to fibrotic deposition and that SIX1 levels are increased in adipocytes by day 7 of bleo treatment.

### Transgenic Six1 deletion attenuates bleomycin-induced skin fibrosis.

**Figure 5:**
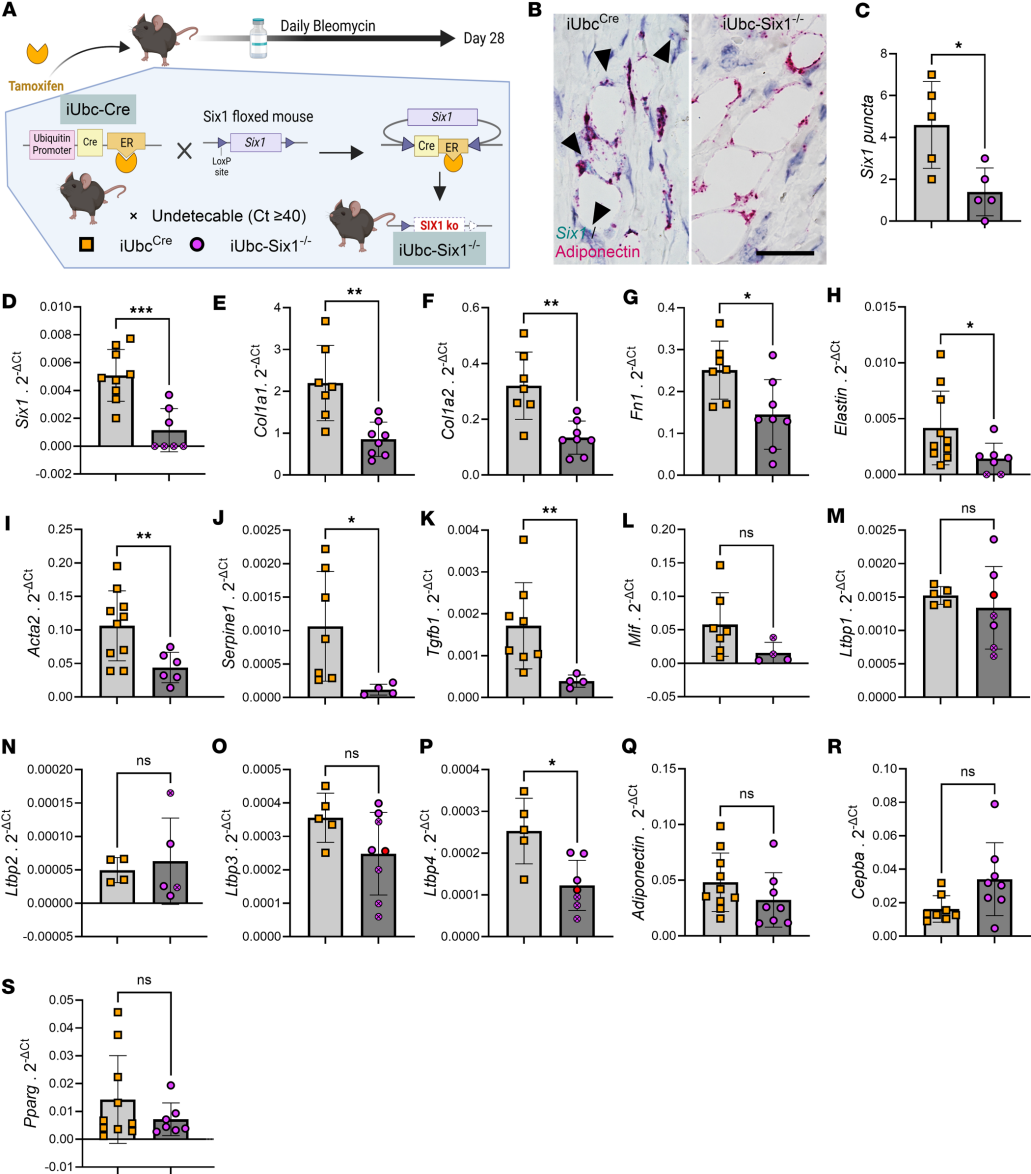
Inducible global deletion of Six1 inhibits fibrotic gene expression in s.c. bleomycin-treated mice. (**A**) Schematic representation of experimental design (created with BioRender). Following i.p. tamoxifen administration, iUbc^Cre^ and iUbc-Six1^–/–^ were given 28 days of s.c. vehicle (PBS) or bleo. (**B** and **C**) Representative dual *Six1 RNAscope* (teal) and *adiponectin* (magenta) from bleomycin iUbc^Cre^ and iUbcSIX1^–/–^ treated mice. Corresponding *Six1* puncta quantification. Arrowheads point at *Six1* signals. Scale bar: 20 μm. (**D**–**S**) Transcript expression levels for sine oculis homeobox homolog 1 (*Six1*, **D**),collagen 1a1 (*Col1a1*, **E**), collagen 1a2 (*Col1a2*, **F**), fibronectin (*Fn1*, **G**), elastin (**H**), actin α 2 (*Acta2*, **I**), serpin family E member 1 (*Serpine1*, **J**), TGF-β1 (*Tgfb1*, **K**), macrophage migration inhibitory factor (*Mif*, **L**), latent TGF-β binding protein 1 (*Ltbp1*, **M**), *Ltbp2* (**N**), *Ltbp3O* (**O**), *Ltbp4* (**P**), *adiponectin* (**Q**), CCAAT enhancer binding protein α (*Cebpa*, **R**), and peroxisome proliferator activated receptor γ (*Pparg*, **S**) at day 28 of bleomycin treatment. Expression was normalized to 18s rRNA. *P ≤ 0.05, **P ≤ 0.01, and ***P ≤ 0.001 refer to an unpaired *t* test for **C**–**S**. Each individual plot represents a biological *n*. *n* = 3–8.

**Figure 6:**
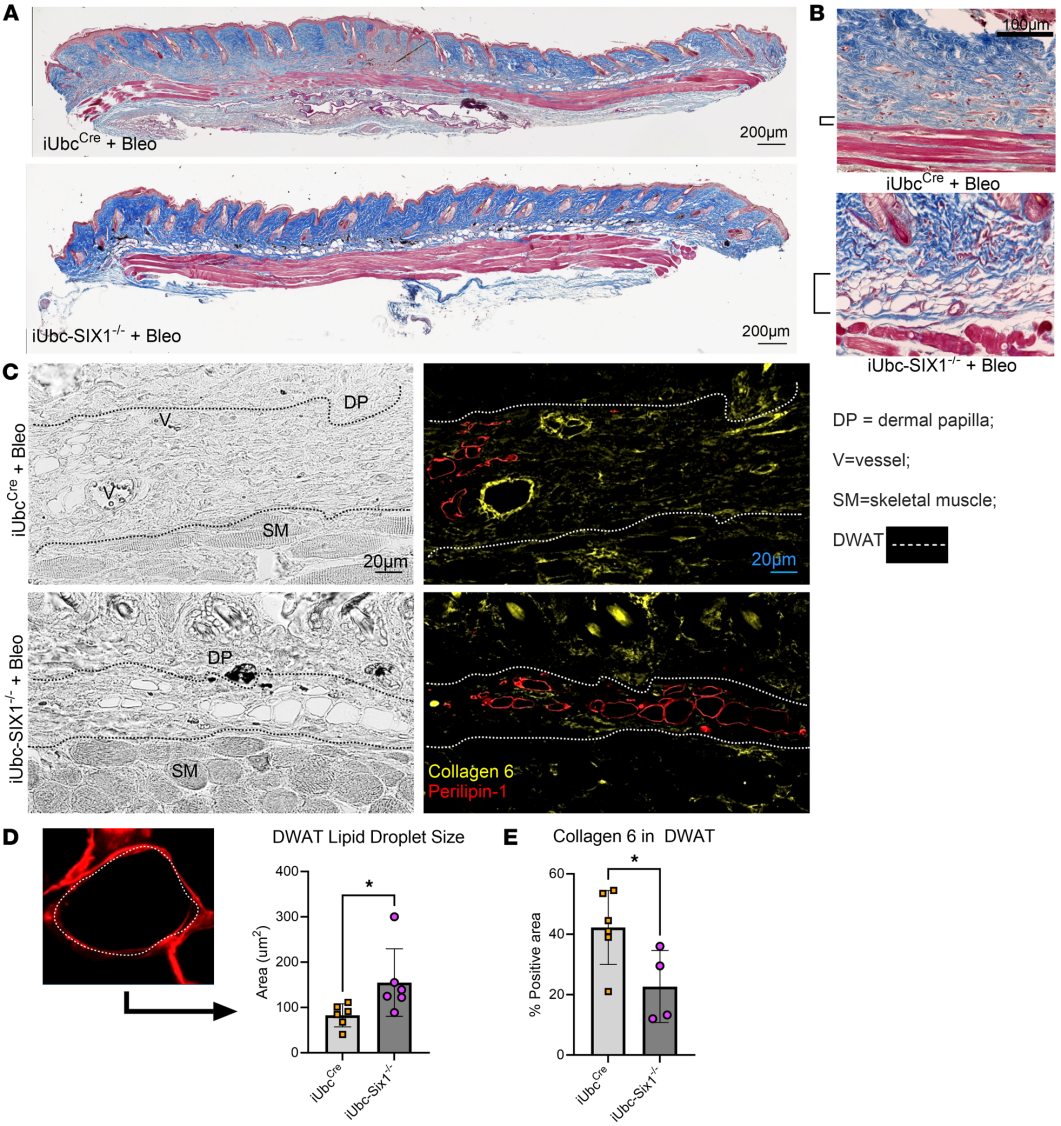
Global *Six1* deletion prevents lipolysis and collagen 6 accumulation induced by s.c. bleomycin. (**A**) Masson’s trichrome staining of skin biopsies from iUbcCre and iUbc-Six1^–/–^ mice treated with 28 days of s.c. bleo. (**B**) High-magnification Masson’s trichrome images of DWAT area marked by square bracket. (**C**) Representative brightfield (left panels) and dual immunofluorescence staining for collagen 6 (yellow signals) and perilipin 1 (red signals) in skin from 28-day bleo injected iUbc^Cre^ and iUbc-Six1^–/–^ mice (right panels). (**D**) Quantification of droplet size of perilipin 1-positive adipocytes in the DWAT. Left: High-magnification (100×, zoomed) image of lipid droplet within DWAT stained with perilipin 1. (**E**) Quantification of collagen 6 as percent positive area in the DWAT. *P ≤ 0.05 refer to an unpaired *t* test. Each individual plot represents a biological *n*. *n* = 4–6. V denotes vessels; SM denotes skeletal muscle; DP denotes dermal papilla. Scale bar: 200 μm (**A**), 50 μm (**B**), and 20 μm (**C**).

We next investigated whether genetic inhibition of *Six1* could prevent skin fibrosis. After a 28-day course of s.c. bleo to induce an end-stage fibrosis phenotype, the affected skin of tamoxifen-treated mice with (iUbc^Cre^) and without the *Six1* allele (iUbc-Six1^–/–^) was analyzed by gene expression profiling and histology (Figure 5A). *Six1* depletion in iUbc-Six1^–/–^ skin following tamoxifen was confirmed morphometrically using dual FABP4 IHC and RNAscope for *Six1* (Figure 5, B and C) and by qPCR (Figure 5D). Expression of profibrotic agents *Col1a1*, *Col1a2*, *Fn1*, *elastin*, *Acta2*, *Tgfb1*, and *Serpine1* but not *Mif* was decreased in the skin of iUbc-Six1^–/–^ compared with iUbc^Cre^ mice (Figure 5, E–L). To investigate whether *Six1* modulates latent TGF-β complex components, we measured Latent TGF-β Binding Protein (*Ltbp*) 1–4 transcripts. Only *Ltbp4* but not *Ltbp1–3* was significantly decreased in iUbc-Six1^−/−^ mice (Figure 5, M–P). These data suggest that *Six1* may selectively affect latent TGF-β sequestration/availability through *Ltbp4*. We did not detect changes in expression of adipocyte markers *Adiponectin*, *Cebpa*, or *Pparg* (Figure 5, Q–S). Masson’s trichrome staining of bleo-affected skin showed maintenance of the DWAT layer in iUbc-Six1^–/–^ mice compared with iUbc^Cre^ mice (Figure 6A). *Six1*-deficient mice had more prominent DWAT with lipid-laden adipocytes (Figure 6B). Perilipin 1 immunostaining was used to specifically detect adipocyte lipid droplets (Figure 6C). Adipocyte droplets in iUbc-Six1^–/–^ were significantly larger compared with iUbc^Cre^ mice (Figure 6D). The deposition of collagen 6, which is enriched in adipose tissue, was analyzed using dual immunofluorescence staining with perilipin 1 to identify the DWAT (Figure 6C) (58, 59). iUbc-Six1^–/–^ had lower collagen 6 density in the DWAT compared with iUbc^Cre^ mice (Figure 6E). There was no significant difference in collagen 6 deposition in the dermis or in dermal thickening (data not shown). In summary, whole-body depletion of *Six1* followed by 28 days of s.c. bleo revealed that *Six1* deletion may halt profibrotic gene expression and maintain DWAT in skin fibrosis.

### To determine whether adipocyte-derived SIX1 contributes to dermal fibrosis, we examined the effect of adipocyte-specific deletion of Six1 in the bleomycin-induced skin injury model.

**Figure 7:**
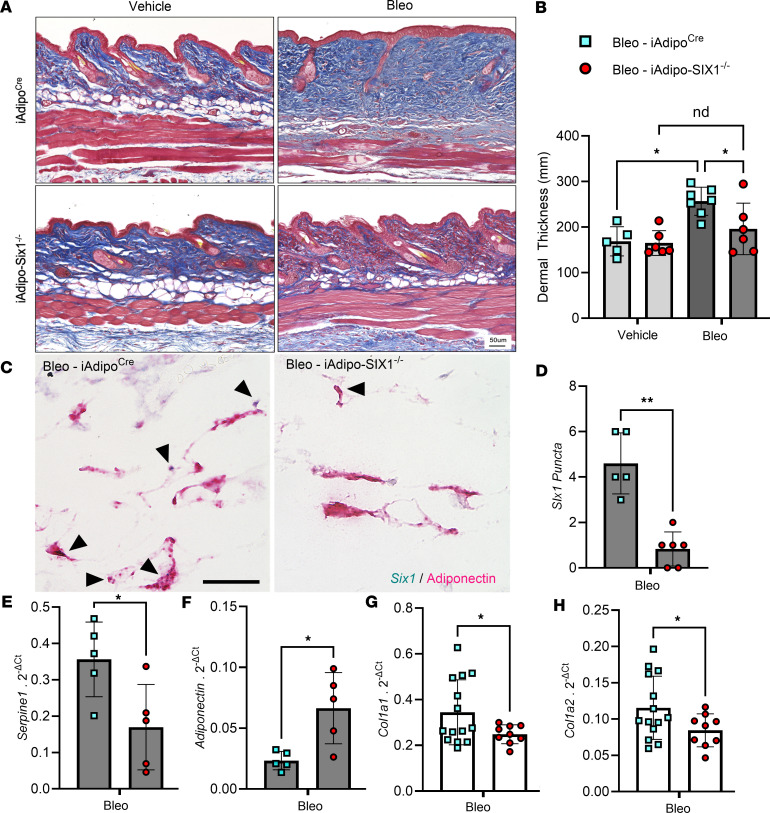
Adipocyte SIX1 deletion prevented bleomycin-induced dermal white adipose atrophy and dermal thickness. (**A**) Representative images of Masson’s trichrome staining from full-thickness skin biopsies. (**B**) Quantification of dermal thickness from Masson’s trichrome staining on day 14 of vehicle (PBS) or Bleo s.c. administration from iAdipo^Cre^ and iAdipo-Six1^–/–^ mice. (**C** and **D**) Representative dual *Six1 RNAscope* (teal) and *adiponectin* (magenta) from bleomycin iAdipo^Cre^– and iAdipo-SIX1^–/–^–treated mice and corresponding *Six1* puncta quantification. Arrowheads point at *Six1* signals. Scale bar: 20 μm. (**E**–**H**) Transcript expression levels for *Serpine 1* (**E**), adiponectin (**F**), collagen 1a1 (*Col1a1*, **G**), or collagen 1a2 (*Col1a2*, **H**), from s.c. bleo-treated iAdipo^Cre^ and iAdipo-Six1^–/–^ mice on day 14 (**E** and **F**) or day 28 (**G** and **H**). *P ≤ 0.05 and **P ≤ 0.01 refer to a 1-way ANOVA with a Bonferroni correction for **B** or unpaired *t* test for **D**–**H**. Each individual plot represents a biological *n*. *n* = 5–13.

To build on our data demonstrating elevated *SIX1* in biopsies from individuals with early SSc and adipose *Six1* expression in a mouse model of dermal fibrosis, when DWAT has begun to atrophy, we next investigated the potential role of adipocyte-specific Six1 in early disease. We developed a transgenic mouse model to knock out *Six1* in cells with an active *Adipoq* promoter after tamoxifen treatment, conferring adipocyte-specific *Six1* depletion in adult mice (48). We challenged mice with s.c. vehicle or bleo for 14 days, as we found this duration of bleo induced DWAT atrophy before late fibrosis is established. Gene expression and histological analyses were performed to determine the contribution of adipocyte *Six1* in early events in skin fibrosis. Masson’s trichrome staining and dermal thickness measurements revealed a thinner dermis in iAdipo-Six1^–/–^ mice after s.c. bleo compared with iAdipo^Cre^ mice (Figure 7, A and B), demonstrating how adipocyte-specific *Six1* deletion prevented dermal thickening compared with *Six1*-competent mice. *Six1* deletion was assessed morphometrically in dual RNAscope for *Six1*, and adiponectin revealed reduced *Six1* puncta in bleo-treated (Figure 7, C and D) iAdipo-Six1^–/–^ mice. Gene expression analysis revealed reduced *Serpine1* but elevated *Adiponectin* expression on day 14 of bleo exposure in iAdipo-Six1^–/–^ versus control iAdipo^Cre^ mice (Figure 7, E and F). Consistent with improved dermal thickness at day 14 of BLEO, we report reduced *Col1a1* and *Col1a2* following s.c. bleo in iAdipo-Six1^–/–^ versus control iAdipo^Cre^ mice (Figure 7, G and H).

**Figure 8:**
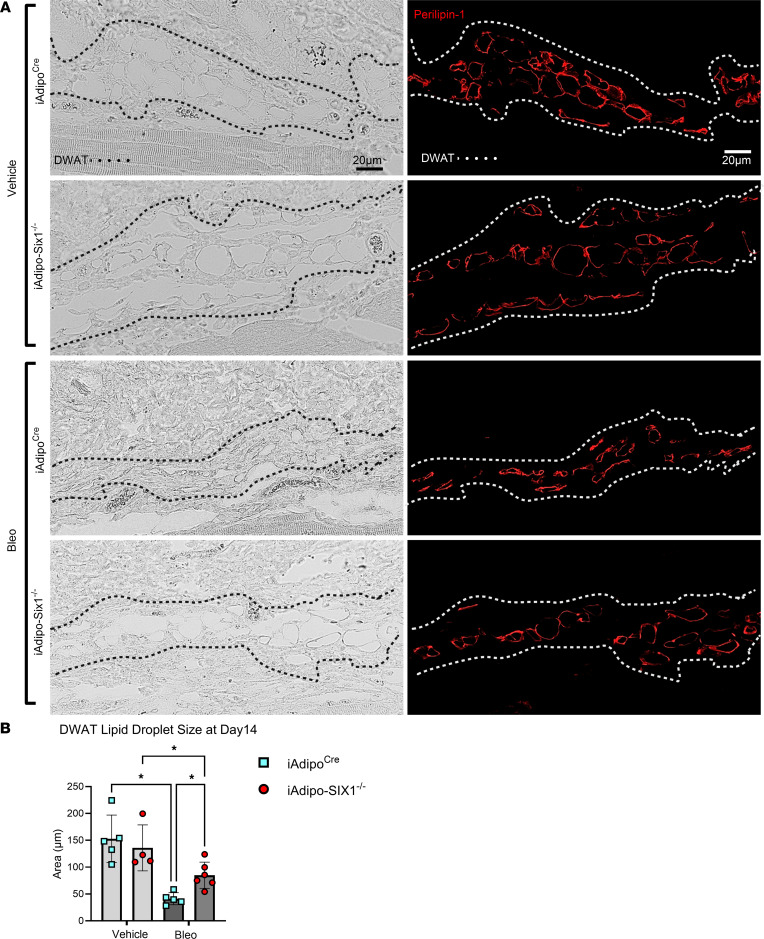
Lipid droplets are preserved in bleomycin-treated adipocyte-Six1–deficient mice treated with bleomycin. (**A**) Representative images of immunofluorescence staining for perilipin 1 in skin from iAdipoc^Cre^ and iAdipo-Six1^–/–^ mice treated with 14 days of s.c. vehicle (PBS) or bleo. Left: bright-field image. Right: immunofluorescence for perilipin (red signals) images. (**B**) Quantification of droplet size of perilipin-positive adipocytes in the DWAT. *P ≤ 0.05 refer to 2-way ANOVA with multiple comparison employing a Holm-Šidák correction for **B**. Each individual plot represents a biological *n*. Scale bar: 20 μm.

**Figure 9:**
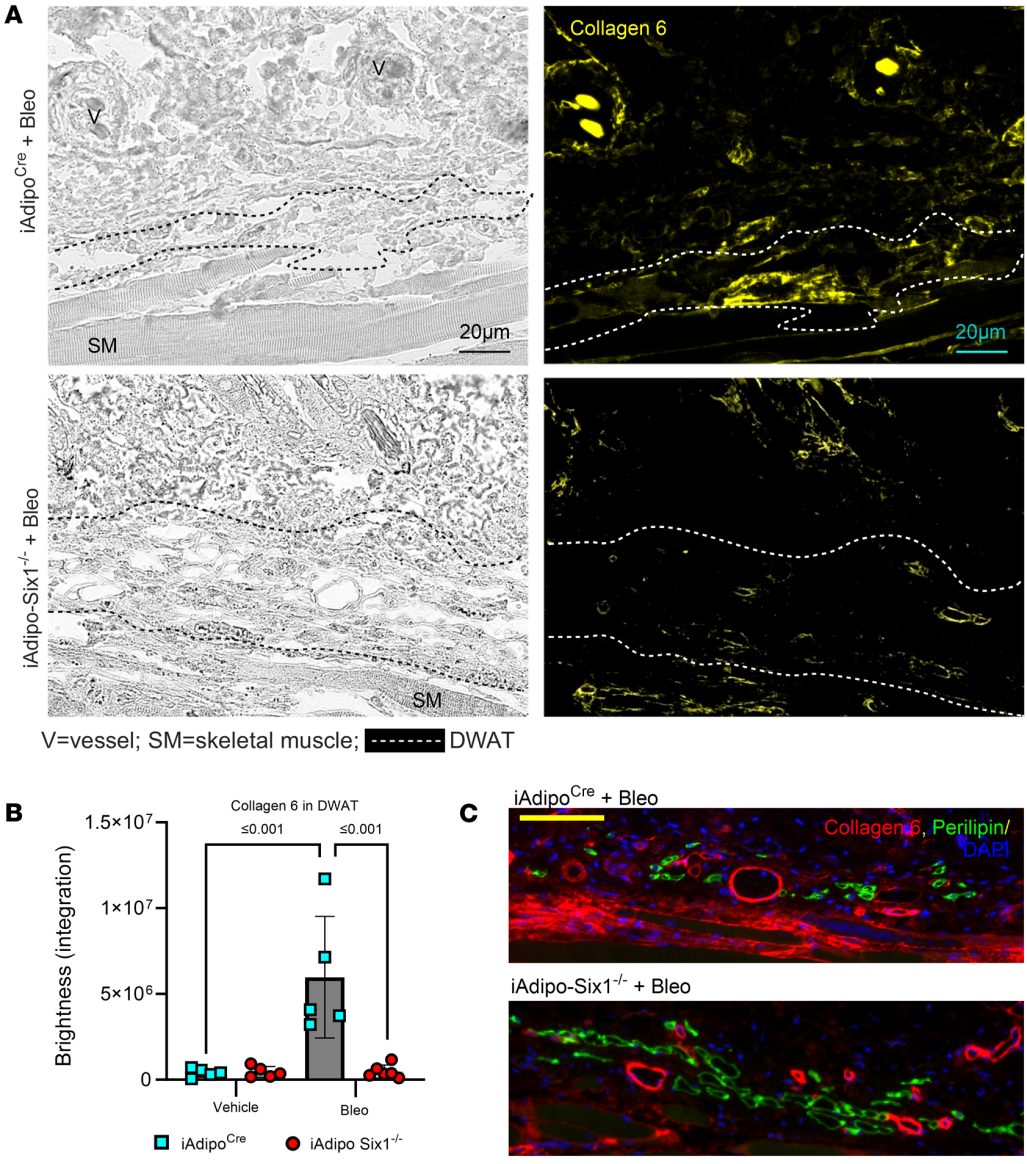
iAdipo^Cre^ -*Six1* KO mice have decreased collagen 6 deposition in the DWAT after 14 days of s.c. bleomycin. (**A**) Representative images of immunofluorescence staining for collagen 6 (yellow) in skin samples from iAdipo^Cre^ or iAdipo-Six1^–/–^ mice treated with 14 days of s.c. bleo. Left: bright-field image. Right: immunofluorescence for collagen 6 (yellow signals). Area within dotted lines represents DWAT. (**B**). Quantification of collagen 6 as brightness intensity within the DWAT. *P* values refer to 2-way ANOVA with multiple comparison, employing a Holm-Šidák correction for **B**. Each individual plot represents a biological *n.* *n* = 5. (**C**) representative dual immunofluorescence for collagen 6 (red signals) or perilipin (green signals) focusing on the DWAT area of either bleomycin-treated iAdipo^Cre^ (upper panel) iAdipoCreSIx1^–/–^ mice (lower panel). V denotes vessels; SM denotes skeletal muscle. Scale bar: 20 μm (**A**) and 50 μm (**B**).

Next, we performed perilipin 1 immunostaining to identify adipocytes in the DWAT and measured intracellular lipid droplet size (Figure 8A). No difference in droplet size was observed in vehicle-treated mice regardless of *Six1* expression. However, adipocyte *Six1* deletion inhibited the reduction in droplet size induced by bleo that precedes loss of adipose tissue (Figure 8B). These findings suggest that *Six1* plays a role in dermal lipid droplet size during the early development of skin fibrosis. To determine whether adipocyte preservation was accompanied by reduced DWAT collagen deposition, we performed immunofluorescence staining to detect collagen 6. While collagens 1, 4, and 6 are abundantly expressed in adipocytes, collagen 6 is the most predominant collagen in fat depots (59); thus, the measurement of such would be expected to have the highest sensitivity for significant changes in the ECM. Deletion of *Six1* in adipocytes significantly reduced DWAT collagen 6 deposition after 14 days of bleo (Figure 9, A and B). This is evident in dual IF for perilipin and collagen 6, demonstrating maintenance of perilipin structures and reduced COL6 in s.c. Bleo-treated iAdiponectinCre-Six1^–/–^ versus iAdiponectin^Cre^ controls (Figure 9C). Adipocyte lipid droplet shrinkage and increased ECM deposition cause dramatic changes to DWAT architecture and function in skin fibrosis. We have here established adipocyte *Six1* as a driver of both lipid droplet size and ECM deposition and as a candidate target for therapeutic intervention.

Collectively, our data demonstrate that *Six1* deletion helps maintain lipid droplet size and ECM deposition in bleo-treated mice. To identify the SIX1 downstream mechanisms, we treated 3T3-L1 fibroblasts with a *siSIX1* (or control siRNA) and an adipocyte differentiation cocktail to promote differentiation to adipose cells. Cells were collected on days 0, 2, 4, 6, and 9. These experiments demonstrated a reduction *SIX1* knockdown on day 4 and 6 by Western blots (Supplemental Figure 1, A and B).

Next, we performed a gene analysis using the nCounter platform targeting fibrotic gene expression on day 6 (D6) of 3T3-treated fibroblasts with and without *siSIX1*. These unbiased experiments and subsequent heat maps for ECM synthesis and TGF-β signaling revealed that knockdown of *SIX1* reduced levels of serine proteinase inhibitor E 1 (SERPINE1) the gene that encodes plasminogen activator inhibitor 1 (PAI-1) (Supplemental Figure 2, A and B). These results also demonstrated reduced *Col4a1*, *Col4a2*, *Col5a1*, *Col5a3*, and *Col6a3* in addition to *Tgfb1*. Analysis of the TGF-β signaling pathway revealed reduced downstream mediators following siSix1 such as *Tgfbr1*, *Crebbp*, or *Furin* (Supplemental Figure 2, A and B). *Serpine1* was selected for further validation based on a volcano plot demonstrating that it was one of the most significantly downregulated genes following siSix1. (Supplemental Figure 3).

**Figure 10:**
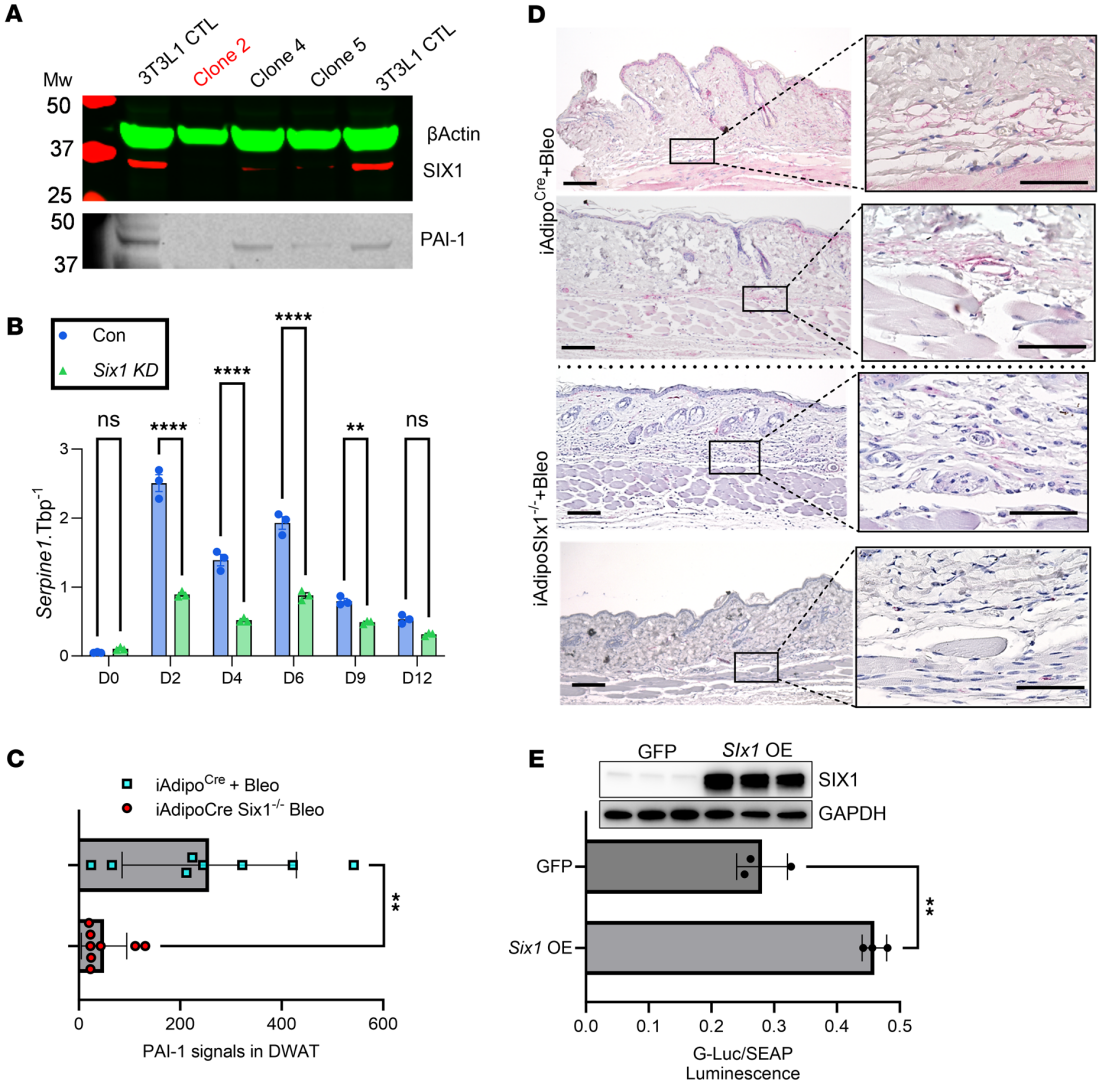
PAI-1 levels are upregulated in bleomycin-induced skin fibrosis and track with SIX1 expression levels. (**A** and **B**) Protein levels for β-actin, SIX1 and PAI-1, and from 3T3-L1 cells treated with a differentiation cocktail for adipocytes and transfected with either control plasmic (blue bars, **B**) or CRISPR/Cas9 *Six1* plasmid (green bars, **A**). (**C** and **D**) Quantification of PAI-1 signals in DWAT from IHC for PAI-1 from bleomycin-exposed mouse skin for from 2 independent iAdipoCre or iAdipoSix1^–/–^ mice. PAI-1 signals are shown in magenta. **P ≤ 0.01, ****P ≤ 0.0001 refer to a 2-way ANOVA with multiple comparison employing a Holm-Šidák correction for **B**. **P ≤ 0.01 refer to an unpaired *t* test for **C**. (**E**) Ratios of Gaussian luciferase/secreted embryonic alkaline phosphatase (G-Luc/SEAP) showing levels of *SERPINE1*-promoter activity from *Six1*OE cells compared with GFP controls at 12 hours. **P ≤ 0.01 refer to an unpaired *t* test for **E**. *n* = 3 (**B** and **E**) and *n* = 8 (**C**). Scale bars represent 20 μm.

To further investigate the role of *Six1* as a regulator of *Serpine1*, we treated mouse 3T3-L1 fibroblasts with a pLenti-CRISPR/Cas9 *Six1* gRNA vector and an adipocyte differentiation cocktail to promote differentiation to adipose cells. Clone 2 was selected for its efficacy at depleting SIX1 levels, consistent with reduced PAI-1 levels (Figure 10A). Cells were collected on D0, D2, D4, D6, D9, and D12, herein *Six1* deletion resulted in reduced Serpine1 levels on D2, D4, D6, and D9 (Figure 10B). The reduction in *Serpine1* expression levels was consistent with reduced PAI-1 signals in DWAT areas in bleo-treated iAdiponectinCreSix1^–/–^ mice versus control Adiponectin^Cre^ mice, assessed morphometrically (Figure 10, C and D). We confirmed *SERPINE1* promoter binding by SIX1 using a full-length h*SERPINE1* promoter Gaussia Luciferase–expressing plasmid. Luciferase activity was significantly increased in *Six1*OE within 12 hours indicating a direct activation of the promoter (Figure 10E). Expression of SERPINE1 was also upregulated 11.5-fold and 3.8-fold in the PRESS and GENISOS cohorts, respectively (Table 1). Although the increased expression of SERPINE1 could reflect secondary processes in fibrosis, our findings support the conclusion that SIX1 upregulation directly promotes SERPINE1/PAI-1 expression as a downstream driver of dermal fibrosis.

Next, to identify whether SIX1 can directly regulate stromal cell fate in dermal fibrosis, we assessed lipid expression levels in mouse 3T3-L1 fibroblasts exposed to adipocyte differentiation cocktail. These studies revealed increased *adiponectin* and *Cebpa* expression levels on D6, D9 (*Cebpa* only) and D12 of CRISPRCas9 deletion of *Six1* (Supplemental Figure 4, A and B). *Fabp4* and *Pparg* followed the same trend with reduced expression in SIX1 KD cells of both of these genes on D2 and D9 (*Pparg* only) and increased expression on D4, D6, and D12 (Supplemental Figure 4, C and D). Fibrogenic gene expression revealed increased *Fn1* expression levels on D0 and D2 but reduced expression on D6 and D9 (Supplemental Figure 4E) in SIX1 KD cell. *Mif* expression levels reduced on D4 and D6 but increased on D12 (Supplemental Figure 4F) following loss of SIX1. *Tgfb1* expression levels were reduced on D2, D9, and D12 but elevated on D6, in SIX1-deficient cells (Supplemental Figure 4G). Intriguingly, the downstream mediator of TGFβ, *Smad3*, did not follow the same trend as *Tgfb* except for D12, where it was also reduced; instead, it was reduced on D0 and elevated on D2 and D9 (Supplemental Figure 4H). Despite the variation in gene expression, these data point to deletion of SIX1 resulting in increased lipid mediators and reduced profibrotic gene expression by D12. This temporal pattern parallels the in vivo sequence, where adipocyte depletion precedes collagen deposition, suggesting that SIX1 orchestrates early transcriptional events determining stromal fate after injury. This is consistent with IF denoting a more adipocyte-like cell following SIX1 KD compared with control cells stimulated with a differentiation cocktail (Supplemental Figure 4I). These in vitro studies suggest that SIX1 acts to repress adipocyte differentiation and favor a more fibroblast-like, profibrotic transcriptional state when treated with a adipocyte differentiation cocktail in vitro.

## Discussion

This study demonstrates the previously undescribed clinical and translational relevance of the developmental transcription factor *SIX1* in adipocyte-mediated skin fibrosis. We identified increased expression of the developmental transcription factor *SIX1* in skin-associated adipose tissue in SSc skin samples from 2 well-described cohorts encompassing 161 patients. The GENISOS cohort contains both lcSSc and dcSSc, allowing us to identify a further increase in *SIX1* in diffuse disease in a large cohort. *SIX1* was also elevated in the skin of individuals with dcSSc diagnosed within 3 years of disease onset, obtained from the PRESS cohort. In agreement with expression data showing enrichment of *SIX1* in dcSSc, *SIX1* skin expression positively correlated with clinical measurements, consistent with worse skin disease extent and severity. Previous work established a s.c. adipose signature in human skin (45, 46, 60). We found this signature to be strongly correlated with *SIX1* expression in both SSc cohorts. It is important to note that a specific dermal adipose signature has not yet been established (56). This observation suggests that, in SSc skin, *SIX1* expression may be associated with genomic changes in adipocyte function. Expression data were supported by histology findings in SSc skin samples from the GENISOS cohort. We reproduced previously reported observations (15, 50) that dermal fat mass declines early and remains atrophic in SSc skin. Using 2 novel transgenic mouse models, we found evidence for a role for *Six1* in lipodystrophy and fibrotic features observed in the s.c. bleo model. Intriguingly, however, only the deletion of *Six1* from adipocytes but not in UBC^Cre^ expressing mice resulted in a reduction of dermal thickening. Possible explanations for this include a more efficient and selective inhibition of *Six1* in adipocytes through the adiponectin Cre system compared with UBC^Cre^ expression or a potential protective response of *Six1* in other cells that could include mesenchymal or inflammatory cells. We propose a working model in which SIX1 helps to drive skin fibrosis by interacting with lipolysis-associated molecular pathways to promote intracellular lipid loss, a critical first step toward transforming a healthy adipocyte to a disease-driving cell type. However, it is also possible for the adipocyte layer to function as a protective element against the development of fibrosis that is lost as tissue atrophies.

We found that *SIX1* expression in SSc skin correlated with genes related to lipid metabolism. We and others have shown that lipid droplets in DWAT are smaller in the s.c. bleo-treated mice (22, 61). Mice with *Six1-*deficient adipocytes (*Six1*^–^ WAs) had larger intracellular lipid droplets than *Six1*^+^ white adipocytes (WAs) after s.c. bleo. Changes in size of unilocular lipid droplets in WAs is a surrogate measurement used to profile an adipose depot as being lipogenic or lipoatrophic (51, 62). A critical step in AMT is the release of free fatty acids into the local tissue environment, a process that permits the transition of a lipid droplet–containing adipocyte into a precursor cell (20). AMT is a fluid process involving dynamic and complex changes in cellular phenotypes and gene expression (24, 63). Dynamic control of lipid storage is required for differentiation and transdifferentiation (47, 64, 65). We found *Six1* to be expressed in mouse DWAT after just 7 days of bleo. While the majority of *Six1*^+^ cells were also positive for *Adipoq*, we observed a minority of *Six1*^+^*Adipoq*^–^ cells: cells other than lipid-laden adipocytes. *Adipoq*^–^ cells in the stroma vascular fraction (SVF) include diverse cell types (66). Furthermore, *ADIPOQ* was highly correlated with *SIX1* in SSc skin. Given the exclusivity of *Six1* expression to the DWAT, we propose that these cells most likely represent those of adipocyte lineage. These studies and the known capacity of SIX1 to promote transdifferentiation (38, 67) point at a role for SIX1 in mediating AMT. In line with this, our in vitro findings provide mechanistic insight into our in vivo observations showing that SIX1 deletion maintains DWAT and adipocyte barrier following fibrotic injury. In cultured 3T3-L1 fibroblasts, loss of SIX1 enhanced adipogenic differentiation, as evidenced by increased expression of *Adiponectin*, *Cebpa*, *Pparg*, and *Fabp4* and reduced expression of profibrotic mediators including *Fn1*, *Mif*, *Tgfb1*, and *Smad3*. This pattern mirrors the phenotype observed in SIX1-deficient mice, where adipocytes were preserved and fibrotic remodeling was attenuated after bleo treatment. Together, these data support a model in which SIX1 promotes AMT by repressing adipogenic transcriptional programs and activating fibrogenic signaling. Deletion of SIX1 prevents this reprogramming, thereby stabilizing adipocyte identity and preserving the DWAT barrier. Given the observed modulation of *Mif* and PAI-1 expressions, SIX1 may also regulate paracrine signaling between adipocytes and fibroblasts, reinforcing profibrotic activation within the dermal microenvironment. To further uncover the mechanism that leads to SIX1-mediated paracrine dermal fibrosis, we turned to a nonbiased approach using the nCounter platform. Here siRNA deletion of *Six1* in 3T3-L1 cells inhibited ECM components such as *Col6a3*, consistent with our IHC for COL6A and other collagens such as *Col4a1*, *Col4a2*, *Col5a1*, and *Col5a3* in addition to *Tgfb1*. In line with previous studies linking *Mif* (30), *Tgfb1* (67), and *Pparg* (43) as targets of SIX1, our studies demonstrated that these mediators we altered following SIX1 deletion using CRISPRCas9. To further explore upstream pathways that may link SIX1 to fibrotic remodeling, we examined expression of *Ltbp* family, which regulates extracellular sequestration and activation of latent TGF-β complexes (68). Among these, only *Ltbp4* was significantly reduced in bleo-iUbc-Six1^–/–^ mice. Because LTBP4 participates in matrix tethering and bioavailability of latent TGF-β, its selective downregulation may attenuate TGF-β activation in the fibrotic niche. These findings raise the possibility that SIX1 modulates dermal fibrosis in part through regulation of latent TGF-β signaling. Since LTBP4 regulates extracellular sequestration and activation of latent TGF-β complexes (69), its reduction in SIX1-deficient mice may contribute to attenuated TGF-β activation and diminished fibrotic remodeling

A common mediator that was altered in both heatmaps was *Serpine1*, the gene encoding for PAI-1. These results reveal reduced *Serpine1* following *Six1* deletion by CRISPRCas9, a result that was validated by qPCR and Western blots and by IHC in skin sections from bleo-treated mice, revealing reduced PAI-1 signals in mice lacking *Six1* in adipocytes. This was further confirmed with a luciferase assay demonstrating that SIX1 is able to bind to the Serpine1 promotor. These results are significant since elevated PAI-1 has been shown to be elevated in skin lesions from patients with SSc (70, 71) and its inhibition improved dermal inflammation and fibrosis in bleo-treated mice (71). These findings suggest SIX1 as upstream from PAI-1 and as a mediator that predisposes adipocytes to a profibrotic phenotype.

Although SIX1 expression was most prominent in dermal fibroblasts, increased SIX1 expression was also observed in macrophage-like and endothelial-like cells in both the GENISOS and PRESS cohorts. The functional significance of SIX1 in these nonfibroblast populations remains to be determined. It is conceivable that SIX1 contributes to profibrotic signaling through modulation of cytokine or angiogenic pathways, consistent with its described roles in other systems (72, 73). Although SIX1 expression was strongest in fibroblast and perivascular clusters, its induction across stromal compartments suggests a broader contribution to the activated dermal niche in SSc.

Targeted therapeutics in SSc are limited as a result of our fragmented understanding of disease mechanisms (74). Two medications, nintedanib and tocilizumab, have been approved by the Food and Drug Administration (FDA) for SSc-related interstitial lung disease (75). However, neither of these drugs or others are FDA approved for SSc skin involvement. SIX1 has been shown to be a potential therapeutic target in pulmonary fibrosis (30). We proposed that SIX1 might also play a role in skin fibrosis. We have shown that genetic deletion of *Six1* is able to reduce dermal adipose tissue atrophy and fibrotic changes in a rodent model of skin fibrosis. These studies demonstrate that adipose tissue homeostasis not only has antifibrotic effects, but that its preservation is a potential therapeutic approach for skin manifestations in SSc. Although our data support a primary role for SIX1 in adipocyte fate determination, its regulation of inflammatory mediators such as MIF suggests that SIX1 may also influence immune-stromal interactions contributing to sustained fibrosis. Together, these findings suggest that pharmacological inhibition of SIX1, or its downstream mediator PAI-1, may represent a novel strategy for preventing or treating dermal fibrosis in SSc.

## Methods

### Sex as a biological variant

In our human and mouse studies, female and male sexes were examined, and no significant differences were observed between sexes.

### Study populations

#### GENISOS cohort.

The prospective cohort study, GENISOS, is a collaboration between UTHealth Houston, The University of Texas Medical Branch at Galveston, and the University of Texas Health Science Center at San Antonio, Texas, USA. All participants met the diagnosis of SSc according to the American College of Rheumatology (ACR) preliminary classification (76). Details of recruitment and selection criteria have been previously published (77). As described in ref. 78, full-thickness skin biopsies (forearm or back) were collected under local anesthesia. Total RNA was extracted using TRIzol reagent (Invitrogen) followed by RNeasy column purification (Qiagen). RNA integrity was confirmed (RIN > 7) before hybridization to the Illumina HumanHT-12 v4 Expression BeadChip.

Individuals with dcSSc and lcSSc are enrolled within 5 years of disease onset, defined as the first non-Raynaud’s symptom.

The mRSS was calculated by a board-certified rheumatologist with extensive experience in the assessment of SSc skin (79). The mRSS is determined by assessment of the skin thickness of 17 body areas by physical examination. The mRSS serves as a surrogate for disease activity, severity, and mortality in patients with SSc (80). Healthy control individuals were enrolled to serve as controls. SSc-affected individuals and controls were matched at a ratio of 3:1 based on age, sex, and ethnicity. Gene expression analysis from SSc-affected skin and skin from controls has been previously described (78). Raw probe-level intensities were imported into BRB-ArrayTools v4.7.1, log_2_ transformed, and quantile normalized. Probes with > 20 % missing values or mean signal below background were excluded prior to differential expression analysis. Briefly, global gene expression is assessed using the Illumina HumanHT-12 bead array. Raw data were analyzed with BRB ArrayTools. 113 SSc-affected individuals, and 44 unaffected controls had available *SIX1* expression levels.

#### PRESS cohort.

The PRESS cohort is a multisite observational cohort of individuals with dcSSc enrolled within 3 years of the onset of the first non-Raynaud’s symptom (9). All participants fulfill the 2013 ACR/European League Against Rheumatism (EULAR) classification criteria for SSc (81). RNA-seq data from the skin of PRESS participants and controls, previously utilized by our group (46), was queried for expression of *SIX1*. Forty-eight SSc-affected individuals and 33 controls had available *SIX1* expression levels. RNA-seq libraries were generated from total RNA using the Illumina TruSeq Stranded mRNA kit and sequenced (2 × 100 bp) on a HiSeq 2500. Reads were aligned to the human GRCh38 reference genome using STAR v2.7, and raw counts were normalized and tested for differential expression with edgeR (82). The R Bioconductor package edgeR6 analysis was utilized to identify differentially expressed transcripts between patients with SSc and healthy controls with a FDR cutoff of 0.05 and fold change cutoff of >1.5 or <0.67

### Cell type–specific expression signatures

Cell type–specific expression signatures were originally developed as previously described (60) and have been utilized by our group (46). A “cell-type specific signature score” denotes a set of genes for which expression in a given cell type is notably higher than expression in the other evaluated cell types. Signature matrices were derived from previously published SSc skin transcriptomic datasets (83, 84). For each sample, the mean fold-change (SSc versus control) across 125 signature genes defined the cell-type score. Spearman’s rank correlations were computed between each score and SIX1 expression to assess lineage association. The numerical value of each score was calculated based on fold-change estimates (SSc versus control) for 125 “signature genes” of a given cell type. The methodology used has been described in detail (60, 78, 85); for gene expression analysis, data were imported into BRB-ArrayTools as processed signal values. Values were excluded if the mean signal was not significantly greater than the background. Values were then log_2_ transformed, followed by quantile normalization. Genes with > 20% missing values across arrays were filtered out. The remaining gene values were used for analyses.

### Pathway analysis

Herein, we used the same protocol described previously (60, 78, 85), genes that were differentially expressed on average in SSc compared with control at a FDR <0.05 were uploaded to Ingenuity Pathway Analysis (Qiagen). The reference set was Human Genome CGH 44 K; only experimentally observed direct and indirect relationships were included. Canonical pathway and upstream-regulator analyses were performed at FDR-adjusted *P* < 0.05 and confirmed with FDR < 0.1.

### Correlation analyses

The correlation between signature score and *SIX1* expression was evaluated for each sample, and then the mean Spearman’s rank correlation coefficient was reported. Individual gene correlations were analyzed by Spearman’s rank correlation. Correlation coefficients are reported as *r*. All correlation analyses were performed in R v4.2 using the Hmisc package; significance was defined as 2-tailed *P* < 0.05. Plots were generated in ggplot2.

### Functional annotation of all DEGs in PRESS

All DEGs in the skin biopsies of PRESS cohort participants with SSc were input into The Database for Annotation, Visualization and Integrated Discovery (DAVID) for functional annotation (https://davidbioinformatics.nih.gov/). Analyses were conducted in DAVID v6.8 (86) using the full human genome as background. Biological processes and molecular functions were considered enriched at Bonferroni-adjusted *P* < 0.05. A Bonferroni cut-off of 0.5 was used to determine significantly enriched biological pathways.

### Animal studies

All studies were reviewed and approved by UTHealth Houston Animal Welfare Committee (AWC-19-0029, AWC-22-0028). Six- to 8-week-old male and female C57BL/6J mice were used for experiments with WT mice. Mice with dorsal skin in the telogen phase of hair cycle were used. Regions of skin in the anagen phase were excluded. When possible, littermates were equally distributed between groups. Group sizes were determined by power analysis (α = 0.05, power = 80%) and restricted to telogen-phase skin, as exclusion of anagen-phase regions reduces biological variability and provides reliable statistical power with fewer animals (87).

Detailed experimental procedures are available in the supplement, including a complete list of SYBR green primers is listed in Supplemental Table 1.

### Statistics

Prism software (v9.0; GraphPad or higher) was used for all statistical analyses. ROUT outlier test was performed on all datasets. Outliers were excluded if FDR was greater than 1%. Two-tailed *t* test with Welch’s correction was used for 2-group comparisons. Two-way ANOVA with multiple comparisons and correction using the Holm-Šidák method was used for 3 or more groups. Detailed statistical analysis for each experiment is shown in the figure legends.

### Study approval

We acknowledge PRESS and GENISOS investigators for their contribution to sample and data collection. Animal experiments were approved by UTHealth Houston Animal Welfare Committee. The study was approved by the IRB of all participating sites, and written informed consent was obtained from all individuals.

### Data availability

The datasets during and/or analyzed during the current study are available from the corresponding author upon reasonable request.

## Author contributions

NW, AS, and HKQ conducted experiments, acquired data, analyzed data, and wrote the manuscript. TWM, SC, MW, LR, RAG, ML, BS, WB, HL, AD, and MK conducted experiments, acquired data, and analyzed data. WRS analyzed data and provided research material. MAA, YY, and WJZ performed bioinformatic analyses, and ARF and HK ran the nCounter experiments. All authors read and approved the manuscript for submission.

## Funding support

Rheumatology Research Foundation Future Physician Scientist Award (NW)

- NIH (NHLBI) R01HL138510, R01HL157100 (HKQ)
- National Scleroderma Foundation Established Investigator Award (HKQ)
- NIH/NIAMS R01AR073284 (Assassi-Mills)
- NIH/NIAMS R01AR081280 (Assassi-Wu)
- NIH/NIAMS K08AR081402 (BS)
- Rheumatology Research Foundation Investigator Award (BS)
- NIH 1UL1TR003167 (WJZ)
- DoD W81XWH-22-1-0164 (WJZ)

## Supplementary Material

Supplemental data

Unedited blot and gel images

Supporting data values

## Figures and Tables

**Table 1:**
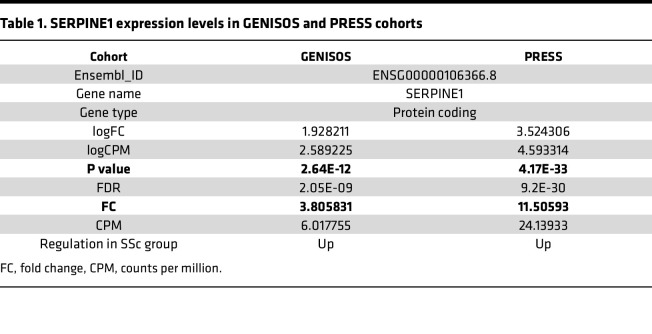
SERPINE1 expression levels in GENISOS and PRESS cohorts

## Version 1 02/19/2026

In-Press Preview

## Version 2 04/08/2026

Electronic publication
