# Supplementary material for: Adipocyte-specific deletion of sine oculis homeobox homolog 1 inhibits lipolysis and reduces skin fibrosis
Source: JCI Insight. 2026 Feb 19;11(7):e181427. doi: 10.1172/jci.insight.181427 (PMC13135399; doi:10.1172/jci.insight.181427)
Supplement: Supplemental data [file jciinsight-11-181427-s012.pdf]

Supplementary Methods

Subcutaneous bleomycin model

The shaved dorsum of isoflurane-anesthetized mice was injected subcutaneously with phosphate-buffered saline (PBS; vehicle) or 0.1 Units/ml bleomycin sulfate USE ([bleo]; Teva Pharmaceuticals) diluted in PBS. For up to 28 days, 0.02 Units of bleo per mouse per day was distributed between two sites, upper and lower dorsal (100µl per site) 6 days per week, as previously described 59. The upper dorsal section was utilized for RT-qPCR, and the lower dorsal for histological analyses. The day following the last injection, mice were euthanized with CO2 followed by cervical dislocation. Any mice in the anagen stage of the hair cycle were excluded from final analyses.

Transgenic mouse models

Tamoxifen-inducible ubiquitous Six1 knock-out mice (iUbc-Six1-/-) were generated by cross-breeding B6.Cg-Ndor1Tg(UBC-cre/ERT2)1Ejb/1J (iUbcCre) (Jackson Labs Cat No. 007001; a gift from lab of Holger Eltzschig, McGovern Medical School, Houston, Texas, USA) and Six1loxP/loxP (a gift from Pascal Maire, Inserm U1016, Institut Cochin. 75014 Paris, France). Tamoxifen-inducible adipocyte-specific Six1 knock-out mice (iAdipo-Six1-/-) were generated by cross breeding C57BL/6-Tg(Adipoq-cre/ERT2)1Soff/J (iAdipoCre) (Jackson Labs; Cat No. 025124) and Six1loxP/loxP mice. Six1loxP/loxP mice contain loxp sites within the 5' untranslated region of exon 1 and the intronic sequence between exon 1 and exon 2 of the Six1 gene.60 iUbcCre and iAdipoCre mice express a Cre recombinase-mutated estrogen receptor fusion protein (Cre-ERT2) under transcriptional control of the human Ubiquitin C/Ubc promoter or the mouse Adiponectin/Adipoq promoter, respectively.61 When bound by tamoxifen, Cre-ERT2 translocated from the cytoplasm to the nucleus, targets loxp sites, and efficiently deletes the floxed allele.62 All mouse strains have a C57BL/6 background.

Tamoxifen treatment

Mice were administered tamoxifen (Sigma-Aldrich, Cat# T5648) dissolved in corn oil (20mg/ml) shaken overnight at 37°C. Mice were intraperitoneally administered 75mg/kg tamoxifen in corn oil every 24 hours for 5 days. Mice were then monitored for 7 days prior to the start of the experiment (Protocol from The Jackson Laboratory; https://www.jax.org/research-and-faculty/resources/cre-repository/tamoxifen).

Tissue processing

The dorsum of mice was shaved, depilated with Nair, then cleaned and allowed to air dry. Two 8mm punch biopsies were taken from sites of injection. The inferior biopsy was immediately placed in 10% neutral buffered formalin and stored at 4□C for at least 48 hours and no longer than 1 week. Formalin-fixed tissue was embedded in paraffin. The superior biopsy was cut in half. One half was placed in cold RNAlater (Sigma-Aldrich, Cat# R0901) and stored per manufacturer's instructions. For RNA isolation, frozen tissue

was finely minced and placed in QIAzol Lysis Reagent (Qiagen, Cat# 79306). Tissue was homogenized on ice using the TissueRuptor (Qiagen, Cat# 9002755), and the homogenate was incubated at room temperature for 5 minutes. RNA extraction was performed using the miRNeasy Mini Kit (Qiagen, Cat# 217004) according to the manufacturer's instructions. RNA was eluted and stored at -80□C until use.

Reverse transcription quantitative polymerase chain reaction (RT-qPCR)

RNA quality was measured by bioanalyzer using RNA 600 Nano Assay (Agilent, Part No 5067-1511). RNA with an RNA Integrity Number > 7 was used for downstream experiments. RNA concentration was measured by nanodrop. Reverse transcription was performed using the QuantiTect Reverse Transcription Kit (Qiagen, Cat#205313) with genomic DNA removal. PCR was carried out with iTaq Universal SYBR Green Supermix (Bio-Rad, Cat#1725124). A complete list of SYBR green primers is listed in **Supplementary Table 1**. qPCR was performed using BioRad CFX Opus 384 Real-Time PCR System. Data processing was performed on BioRad CFX Maestro. Expression values are reported as 2-ΔCt (Ctgene of interest – Ct18s rRNA).

Immunofluorescence (IF)

Immunofluorescence of FFPE mouse tissue sections was performed as described63. Five-micron thick formalin fixed paraffin-embedded (FFPE) skin tissue was deparaffinized then heat-based antigen retrieval was performed in high pH Tris-based antigen retrieval buffer (Vector Laboratories, Cat# H-3301). After washing in tris-buffered saline containing 0.1% Tween-20 (TBS-T), tissue was blocked in 2.5% - 10% animal serum for 1 hour at room temperature. Primary antibodies were perilipin 1 (Abcam #ab3526, 1:200), collagen 6 (Abcam #ab182744, 1:100) goat anti-perilipin-1 (1:100; GeneTex cat# GTX89109), aSMA-Cy3 (1:200; Sigma-Aldrich Cat# C6198). Secondary antibodies used: donkey anti-goat Alexa Fluor 488-conjugated IgG (1:300; Invitrogen Cat# A11055) and donkey anti-rabbit Cy3-conjugated IgG (1:300; Jackson ImmunoResearch Cat# 711-166-152). Nuclei were stained with Hoechst 33258 (Invitrogen, 1:2000). Tissue images were acquired with a Carl Zeiss upright Apotome Axio Imager Z1 microscope. Primary antibodies were incubated overnight at 4□C. After washing in TBS-T, tissue was incubated with Alexa Fluor secondary antibody (ThermoFisher Scientific) for 1 hour at room temperature. Autofluorescence was quenched using TrueVIEW Autofluorescence Quenching Kit (Vector Laboratories, Cat# SP-8400-15). Tissue was mounted using ProLong Gold Antifade Mountant with DAPI (Invitrogen, Cat# P36931).

In situ hybridization

RNAScope, an optimized single-molecular in situ hybridization technique 64 was used to detect single RNA transcripts in FFPE human and mouse skin tissue. RNAScope 2.5 HD Assay – RED (Advanced Cell Diagnostics; Cat No. 322360) and RNAScope 2.5 HD Duplex Reagent Kit (Advanced Cell Diagnostics; Cat No. 322430) were used for detection of one or two transcripts, respectively. Complete protocol is available from manufacturer. RNAScope probes are listed in Supplementary Table 2.

Imaging and histological analysis

Histology images were captured on the Keyence BZ-X810 microscope and analyzed using Keyence BZ analyzer software. Adipocyte droplet size was measured by calculating the area within the droplet as outlined by cytoplasmic perilipin 1 staining. Collagen 6 immunofluorescence was quantified as percent positive area of the DWAT or brightness intensity within the DWAT. A macro was created using the Hybrid Cell Count feature to ensure unbiased and consistent quantification parameters. DWAT was defined by the region containing positive perilipin 1 staining and the upper border of the panniculus carnosus. Hair follicles and large vessels were excluded. Four to five non-continuous images per mouse biopsy were used for all histological analyses.

DWAT Quantification

Dermal white adipose tissue (DWAT) was defined as the perilipin-1–positive adipocyte-rich layer extending to the upper border of the panniculus carnosus, as previously described 65,66. Perilipin-stained sections were imaged using a Keyence BZ-X810 microscope, and DWAT area and adipocyte droplet size were quantified using BZ Analyzer software. Collagen VI signal within DWAT was measured as percent positive area using a Hybrid Cell Count macro to ensure unbiased analysis. 4-5 40x images were analyzed per mouse. Hair follicles and large vessels were excluded from quantification. This method is consistent with established approaches for assessing DWAT remodeling in murine dermal fibrosis 22.

SIX1 Puncta Quantification

SIX1 expression was visualized by immunofluorescence or RNAscope in situ hybridization using probes specific to Six1 (ACD #838641 for mouse; #412401 for human). For quantification of Six1 puncta, confocal images were acquired at 40× magnification using a Keyence BZ-X810 microscope. Puncta within nuclei were counted manually or via automated detection in Keyence BZ Analyzer software using a Hybrid Cell Count macro to ensure unbiased quantification. Each punctum corresponded to an individual Six1 transcript or protein focus. Counts were averaged from 3–5 non-contiguous fields per section per mouse. Positive SIX1 signal was defined by co-localization with DAPI (for nuclear staining) and, where applicable, Adiponectin or FABP4 or SMA signal to confirm cell identity. Large vessels, follicles, and auto fluorescent regions were excluded from quantification to minimize background signal.

PAI-1 Quantification

For detection of plasminogen activator inhibitor-1 (PAI-1), mouse skin sections were immunostained and imaged at 40× magnification under brightfield using a Keyence BZ-X800 microscope (Itasca, IL). Representative fields were selected from the dermal and subdermal regions, and staining intensity was quantified using BZ Analyzer software with identical exposure settings across all groups.

In Vitro Cell Culture Studies

Low-passage 3T3-L1 cells (ATCC Cat#CL-173) were thawed and grown to 70% confluency in 10ml of complete pre-adipocyte growth media composed of high glucose Dulbecco's Modified Eagle's Medium (DMEM) containing 4mM L-glutamine (Sigma Aldrich, Cat# D5796) supplemented with 10% calf serum (Colorado Serum Company, Cat#31334), 1% penicillin and streptomycin (Gibco, Cat# 15140-148), and 1mM sodium pyruvate (Sigma Aldrich, Cat# S8636). Cells were washed in PBS and media was replaced every 72 hours. To prevent growth arrest, expanding pre-adipocytes were never allowed to reach greater than 70% confluency. Cells were detached using 0.25% trypsin-EDTA.
CRISPR/Cas9-mediated knockout of Six1 in 3T3-L1 cells was performed. In brief, a pLenti-CRISPR/Cas9 Six1 gRNA vector, with 20-bp target sequence 5′-AGAGCAATATGGGCCACGCCAGG-3′ containing the PAM sequence (underlined) was subcloned into lenti-CRISPR v2 plasmid (Addgene no. 52961) and used to generate the lentivirus to knockout of Six1 in 3T3-L1 cells. To select successfully transduced cells, cells were cultured in DMEM+10%FBS containing 5 µg/mL puromycin for 7 days.

To induce pre-adipocyte differentiation into mature white adipocytes, previously described protocols were utilized63,67 and the cells were allowed to grow until day 9. Control siRNA (Sigma SIC001) or small interfering siRNA for Six1 (Sigma SASI_Mm01_00198105) was transfected to 3T3 cells on days -4, -2 of differentiation to mature fibroblasts. For RNA isolation, 85µl per cm2 of cold Trizol (Invitrogen, Cat# 15596026) was used. For protein isolation, 8µl per cm2 of cold RIPA buffer with protease and phosphatase inhibitors (ProteinBiology, Cat# 78442) was used. For immunoblots, cells were lysed with RIPA buffer (Thermo Scientific) containing Halt Protease and Phosphatase inhibitor (Sigma Aldrich). Samples were loaded in reducing Sodium dodecyl-sulfate (SDS) buffer (BD Biosciences) in 4-20% Tris-Glycine eXtended (TGX) gels (Bio-Rad). Polyvinylidene difluoride (PVDF) membranes were blocked with 5% dry milk in TBST for 1 hr and then incubated overnight with the primary antibody. HRP-conjugated secondary antibody was incubated for 1 hour, and then blots were imaged with SuperSignal West Pico (Thermo Scientific) on a Chemi-Doc Imaging system(Bio-Rad). Antibodies used included: β-Actin(Cell Signaling Technology [CST] 5125S at 1:2000); SERPINE1 (abcam, ab282007 at 1:1000); SIX1 (CST 12891S at 1:1000) and Rabbit HRP (CST 7074S 1:3000).

NanoString nCounter Analysis System:
After RNA extraction from samples, their quality control was done using Agilent TapeStation, and the amount of RNA was quantified with Invitrogen™ Qubit™ RNA High Sensitivity (HS) kit. Later, the samples went through an overnight hybridization process --performed at 65°C in a thermocycler-- where RNA (analytes) were hybridized to nCounter optical barcodes (nCounter® Mouse Fibrosis V2 Panel: NanoString Technologies). After hybridization, samples were processed using the nCounter Pro Analysis System, which rapidly immobilizes and counts samples that have hybridized to

nCounter barcodes. This is an ex-situ digital counting of RNAs. Briefly, on the nCounter Prep Station, hybridized samples get prepared for data collection on the nCounter Digital Analyzer. This preparation includes purification and immobilization in a sample cartridge. On the nCounter Digital Analyzer, hundreds of images are collected for each sample, yielding hundreds of thousands of target molecule counts per sample. After an internal processing of images, the results are RCC (Reporter Code Count) files containing the RNA counts that can be directly processed through the nSolver Analysis Software.

SERPINE1-Luc promoter binding assay.

HEK-293T cells were transfected with either control pcDNA3.1-GFP vector or a pcDNA3.1-hSix1 construct made in our laboratory, along with the hSERBP1promoter–Gaussia Luciferase expression plasmid (HPRM56194-PG04, GeneCopoeia). Supernatants were collected at 12 hours after transfection and assessed for secreted luciferase activity and secreted embryonic alkaline phosphatase, as the transfection control using Secrete-Pair dual luminescence assay kit (GeneCopoeia, LF032). Experiments were run in triplicate wells for all the experimental groups, and supernatant samples that were collected and immediately analyzed.

Supplementary Figure 1. SiSix1 reduces protein SIX levels in 3T3L1

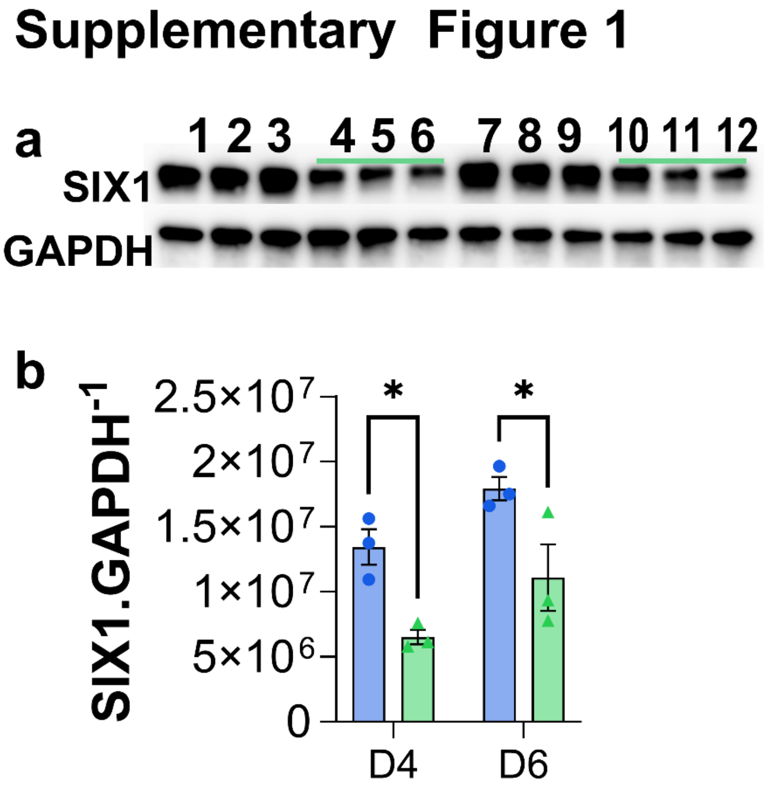

3T3 cells treated with a differentiation cocktail for adipocytes and transfected with either scrRNA (blue bars) or siSix1 (green bars). **a**) western blot for SIX1 or GAPDH lanes 1-3 represent scrRNA transfection on day 4, lanes 4-6 represent siSIX1 transfection on day 4, lanes 7-9 represent scrRNA transfection on day 6 and lanes 10-12 represent siSIX1 transfection on day 6. Densitometries for SIX1 expression relative to GAPDH on days 4 and 6 (**b**).

Supplementary Figure 2. Heat maps from nCounter data identifying Serpine1 as a target. nCounter generated heatmaps for extracellular matrix (ECM) synthesis pathways (**a**) and TGF-β signaling pathway (**b**). Yellow shades represent upregulated genes and blue shades represent downregulated genes. The red arrow points at Serpine1.

Supplementary Figure 2

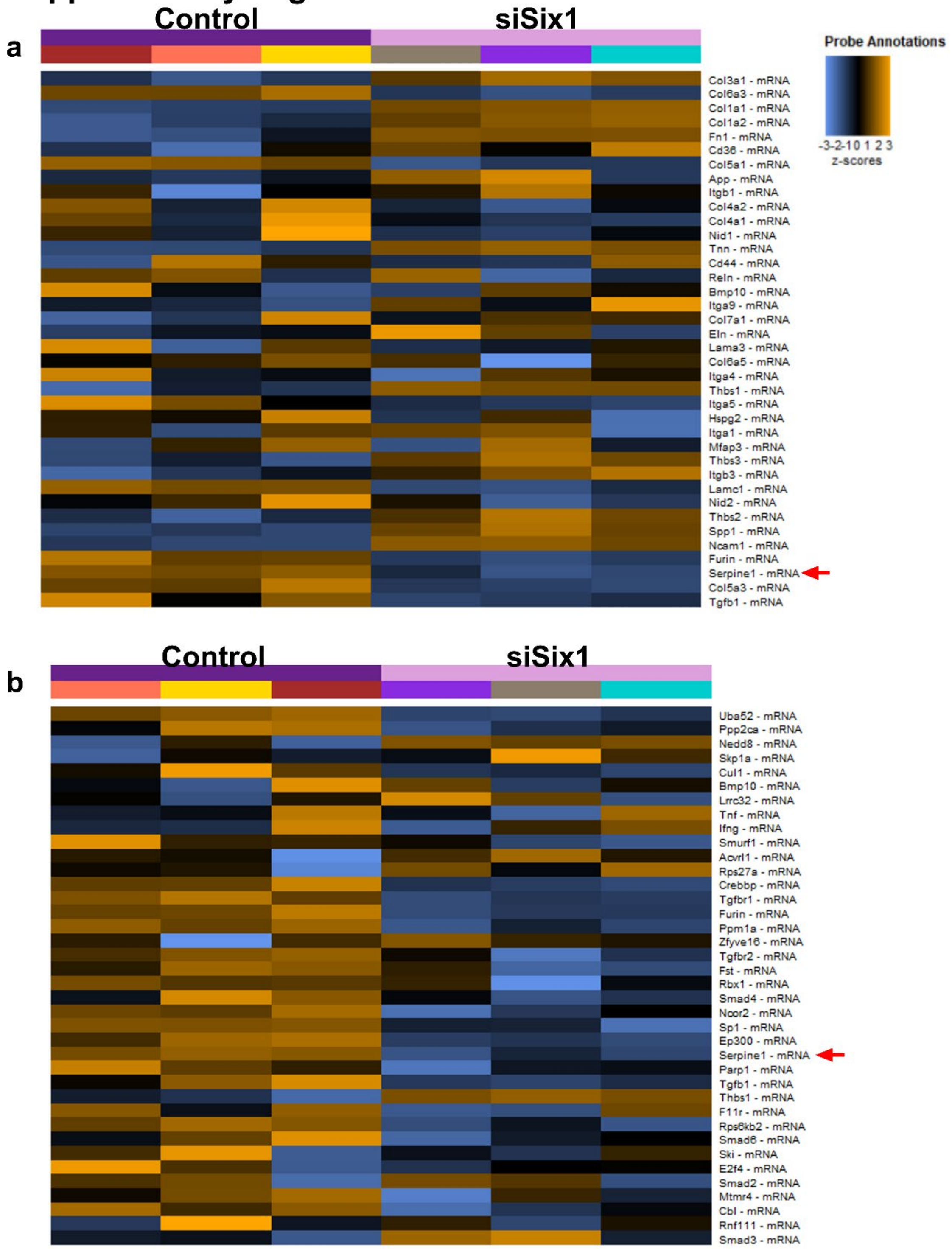

Supplementary Figure 3. Volcano plot from nCounter data identifying Serpine1 as a target following SIX1 deletion.

Supplementary Figure 3

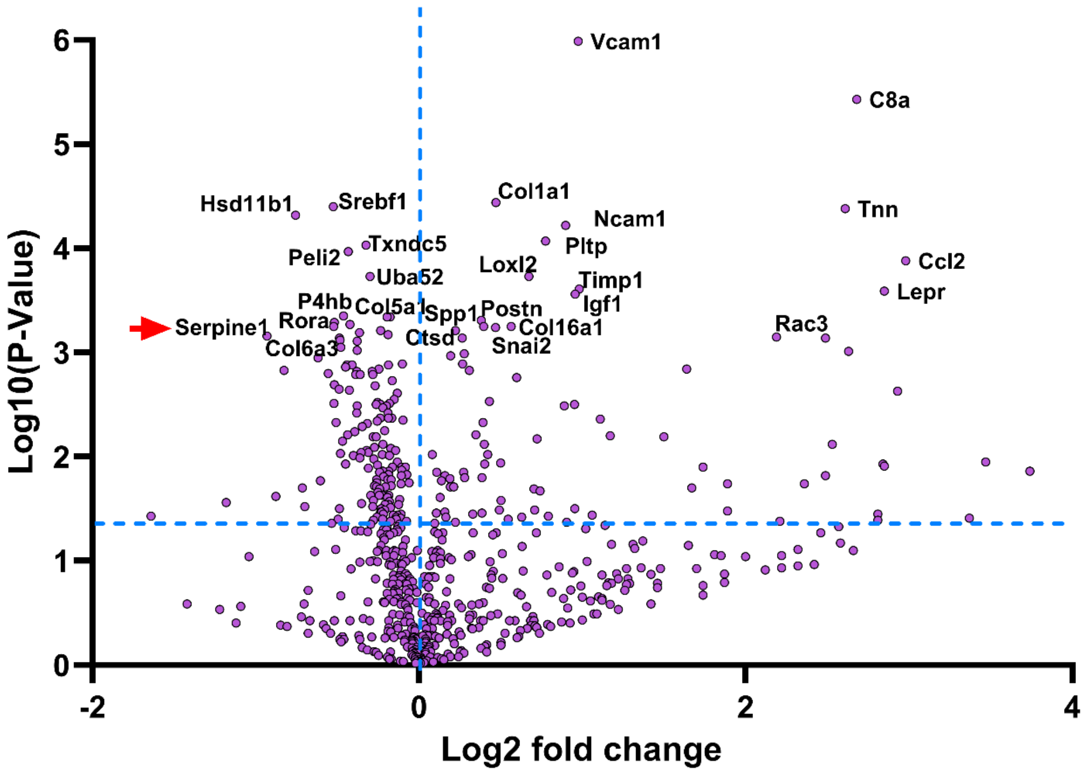

nCounter generated DEG volcano plot from Day 6 scRNA vs siSix1 transfected 3T3 cells treated with a differentiation cocktail to adipocytes. Significant genes are located above the horizontal dotted blue line. The top genes with altered expression are labelled including Serpine 1 identified by a red arrow.

Supplementary Figure 4. Deletion of *Six1* promotes lipid mediators but inhibits profibrotic gene expression

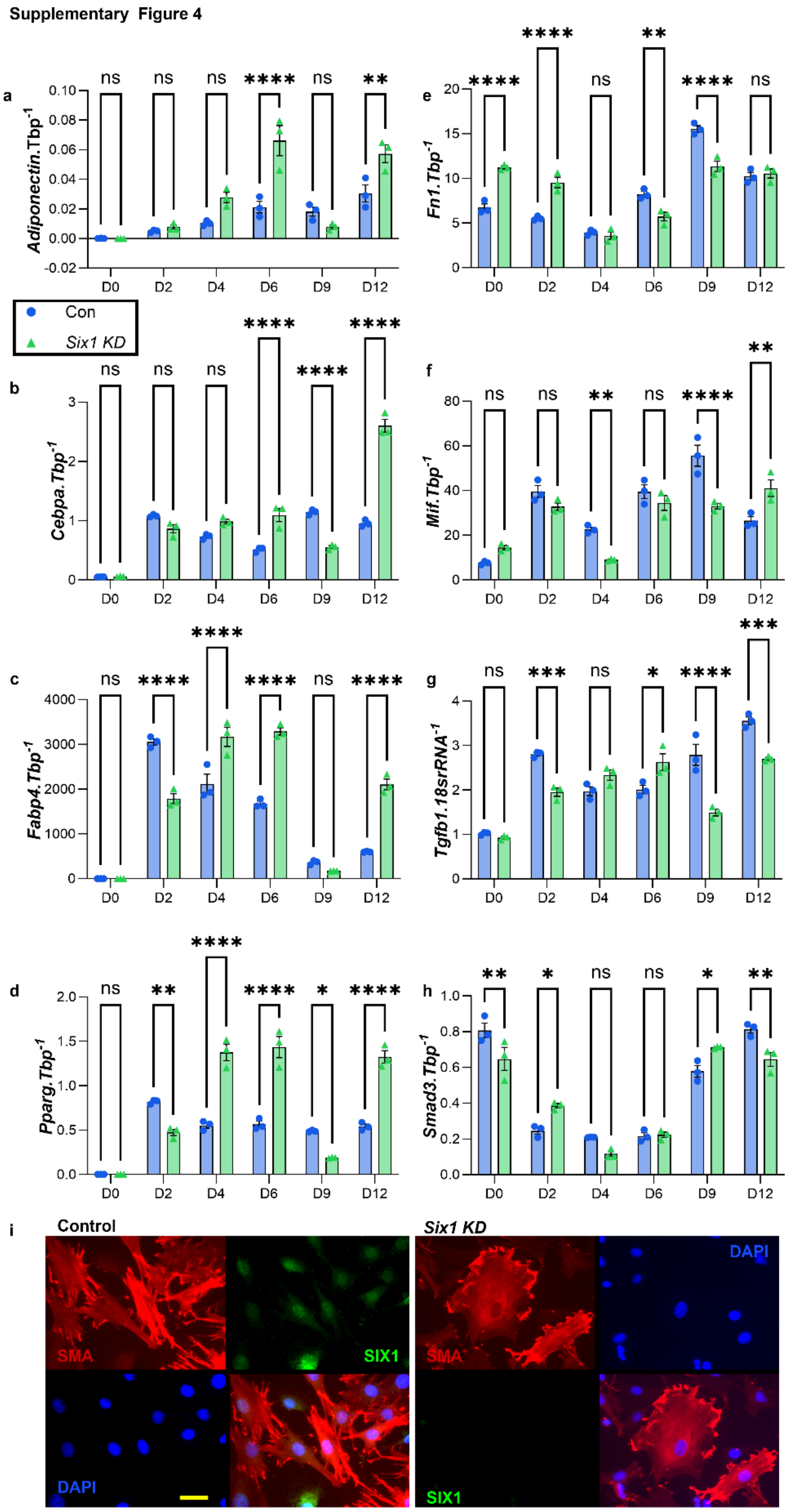

Supplementary Figure 4. CRISPR/CAS9 deletion of Six1 alters cell fate
3T3L1 cells treated with a differentiation cocktail to adipocytes and transfected with either control (blue bars) or CRISPR/CAS9 for *Six1* (green bars). Expression levels for (**a**) *adiponectin*, (**b**) *Cebpa* (**c**) *Fabp4*, (**d)** *Pparg* and (**e**) *Fn1*, (**f**) *Mif,* (**g**) *Tgfb1* and (**h**) *Smad3* from control or CRSPR/Cas9 *Six1* transfected 3T3L1 cells on days (D) 0, 2, 4, 6. 9 and 12 after treatment with the differentiation cocktail. (**i**) Representative imaged on D12 from control or SIX1 KD cells on day 12 denosing SMA (red), SIX1 (green) and counter stained with DAPI (blue siganls). Scale bar represents 50µm. Significance levels * P ≤0.05, ** P ≤0.01, *** P ≤0.001, and **** P ≤0.0001 refer to a Two-way ANOVA with multiple comparison employing a Sidak correction for panels a-h. N=3 per group and timepoint.

Supplementary Table 1: Mouse SYBR green primers used for quantitative polymerase chain reaction

**Transcript name**	**Forward primer sequence (5’ to 3’)**	**Reverse primer sequence (3’ to 5’)**
18s rRNA	GTAACCCGTTGAACCCCATT	CCATCCAATCGGTAGTAGCG
Adiponectin	TGTTCCTCTTAATCCTGCCCA	CCAACCTGCACAAGTTCCCTT
Cebpa	GTCACTGGTCAACTCCAGCAC	CAAGAACAGCAACGAGTACCG
Col1a1	GGTTTCCACGTCTCACCATT	CGGCTCCTGCTCCTCTTAG
Col1a2	AGCAGGTCCTTGGAAACCTT	AAGGAGTTTCATCTGGCCCT
Col6a1	GATGAGGGTGAAGTGGGAGA	CAGCACGAAGAGGATGTCAA
GAPDH	AGGTCGGTGTGAACGGATTTG	TGTAGACCATGTAGTTGAGGTCA
Pparg	GGTGGGCCAGAATGGCATCT	TCTGGGAGATTCTCCTATTGA
Serpine1	TTCAGCCTTGCTTGCCTC	ACACTTTTACTCCGAAGTCGGT
Six1	GAAAGGGAGAACACCGAAAACA	GTGGCCCATATTGCTCTGGA
Tbp	AGAACAATCCAGACTAGCAGCA	GGGAACTTCACATCACAGCTC

Supplementary Table 2: RNAScope probes used for in situ hybridization

Target transcript (RefSeq Accession number)	Target species	Target region	Catalog #
SIX1 (NM_005982.3)	Human	799-2177	ACD; 412401
FABP4 (NM_001442.2)	Human	119 - 826	ACD; 470641-C2
Six1 (NM_009189.3)	Mouse	1932-2819	ACD; 838641
Adiponectin (NM_009605.4)	Mouse	9 - 1233	ACD; 440051-C3

Supplementary Table 3. Top fifty most highly correlated genes with skin *SIX1* in the PRESS cohort by Spearman correlation analysis

Ensembl_ID	Gene symbol	Gene name	*r*	p-value	FDR
ENSG00000166819.10	*PLIN1*	Perilipin 1	0.865	1.30E-19	2.54E-15
ENSG00000187288.9	*CIDEC*	Cell death inducing DFFA like effector C	0.858	5.43E-19	7.07E-15
ENSG00000167676.3	*PLIN4*	Perilipin 4	0.854	1.15E-18	9.13E-15
ENSG00000138207.11	*RBP4*	Retinol binding protein 4	0.854	1.23E-18	9.13E-15
ENSG00000123689.5	*G0S2*	G0/G1 switch gene-2	0.853	1.40E-18	9.13E-15
ENSG00000181092.8	*ADIPOQ*	Adiponectin	0.843	7.82E-18	4.37E-14
ENSG00000165478.6	*HEPACAM*	Hepatic and glial cell adhesion molecule	0.840	1.47E-17	7.19E-14
ENSG00000176485.9	*PLA2G16*	Phospholipase A and acyltransferase 3	0.838	2.01E-17	8.71E-14
ENSG00000079435.8	*LIPE*	Hormone sensitive lipase	0.832	5.58E-17	2.18E-13
ENSG00000184811.3	*TUSC5*	Trafficking regulator of Glut4	0.831	6.58E-17	2.32E-13
ENSG00000175445.13	*LPL*	Lipoprotein lipase	0.830	7.14E-17	2.32E-13
ENSG00000135447.15	*PPP1R1A*	Protein phosphatase 1 regulatory inhibitor subunit 1a	0.819	3.93E-16	1.18E-12
ENSG00000170323.7	*FABP4*	Fatty acid binding protein 4	0.814	9.13E-16	2.55E-12
ENSG00000042286.13	*AIFM2*	Apoptosis inducing factor mitochondria associated 2	0.812	1.15E-15	3.00E-12
ENSG00000185818.7	*NAT8L*	N-acetyltransferase 8 like	0.810	1.61E-15	3.92E-12
ENSG00000174804.3	*FZD4*	Frizzled 4	0.806	2.83E-15	6.51E-12
ENSG00000174697.4	*LEP*	Leptin	0.802	4.51E-15	9.78E-12
ENSG00000129596.4	*CDO1*	Cysteine dioxygenase type 1	0.797	8.75E-15	1.80E-11
ENSG00000173208.3	*ABCD2*	ATP binding cassette subfamily D member 2	0.797	9.23E-15	1.80E-11
ENSG00000005249.11	*PRKAR2B*	Protein kinase camp-dependent type 2 regulatory subunit beta	0.794	1.34E-14	2.49E-11
ENSG00000152270.7	*PDE3B*	Phosphodiesterase 3B	0.794	1.44E-14	2.55E-11
ENSG00000133317.13	*LGALS12*	Galectin 12	0.792	1.71E-14	2.91E-11
ENSG00000168004.8	*PLAAT5*	Phospholipase a and acyltransferase 5	0.792	1.82E-14	2.96E-11
ENSG00000131471.5	*AOC3*	Amine oxidase copper containing 3	0.791	1.90E-14	2.97E-11
ENSG00000134962.6	*KLB*	Klotho beta	0.790	2.20E-14	3.31E-11
ENSG00000165269.11	*AQP7*	Aquaporin 7	0.786	3.88E-14	5.61E-11

ENSG00000164638.9	*SLC29A4*	Solute carrier family 29 member 4	0.785	4.13E-14	5.76E-11
ENSG00000119927.12	*GPAM*	Glycerol-3-phosphate acyltransferase, mitochondrial	0.780	7.65E-14	1.03E-10
ENSG00000169692.11	*AGPAT2*	1-acylglycerol-3-phosphate o-acyltransferase 2	0.777	1.15E-13	1.49E-10
ENSG00000198624.11	*CCDC69*	Coiled-coil domain containing 69	0.773	1.81E-13	2.28E-10
ENSG00000149124.9	*GLYAT*	Glycine-N-acyltransferase	0.772	2.03E-13	2.48E-10
ENSG00000221968.7	*FADS3*	Fatty acid desaturase 3	0.771	2.30E-13	2.72E-10
ENSG00000056998.17	*GYG2*	Glycogenin 2	0.769	2.88E-13	3.31E-10
ENSG00000158571.9	*PFKFB1*	6-Phosphofructo-2-Kinase/Fructose-2,6-Biphosphatase 1	0.768	3.17E-13	3.54E-10
ENSG00000076706.13	*MCAM*	Melanoma cell adhesion molecule	0.763	5.59E-13	6.06E-10
ENSG00000234840.1	*LINC01239*	Long intergenic non-protein coding rna 1239	0.759	8.51E-13	8.89E-10
ENSG00000129675.14	*ARHGEF6*	Rac/cdc42 guanine nucleotide exchange factor 6	0.759	8.65E-13	8.89E-10
ENSG00000123612.14	*ACVR1C*	Activin A receptor type 1c	0.753	1.61E-12	1.61E-09
ENSG00000124253.10	*PCK1*	Phosphoenolpyruvate Carboxykinase 1	0.752	1.79E-12	1.75E-09
ENSG00000009950.14	*MLXIPL*	Mlx interacting protein like	0.747	3.18E-12	3.03E-09
ENSG00000281769.1	*LINC01239*	Long intergenic non-protein coding rna 1239	0.730	1.64E-11	1.52E-08
ENSG00000189367.13	*KIAA0408*	Uncharacterized protein KIAA0408	0.729	1.86E-11	1.68E-08
ENSG00000177666.14	*ATGL*	Adipose triglyceride lipase	0.729	1.89E-11	1.68E-08
ENSG00000158186.11	*MRAS*	Muscle RAS oncogene homolog	0.727	2.24E-11	1.94E-08
ENSG00000277737.2	*FP325317.1*	*uncharacterized transcript*	0.726	2.40E-11	2.04E-08
ENSG00000120049.17	*KCNIP2*	Potassium voltage-gated channel interacting orotein 2	0.722	3.49E-11	2.87E-08
ENSG00000186205.11	*MTARC1*	Mitochondrial amidoxime reducing component 1	0.722	3.53E-11	2.87E-08
ENSG00000171914.13	*TLN2*	Talin 2	0.722	3.66E-11	2.92E-08
ENSG00000119729.9	*RHOQ*	Ras homolog family member Q	0.717	5.67E-11	4.43E-08
ENSG00000151632.15	*AKR1C2*	Aldo-keto reductase family 1 member c2	0.716	5.99E-11	4.59E-08

Supplementary Table 4. Demographic and clinical features of biopsied participants in Figure 2c.

Panel	Age (yrs)	Sex	Race	Disease subtype	Disease duration (yrs)	mRSS	local skin score
Patient 1	60	Male	Caucasian	dcSSc	1.8	19	2
Patient 2	25	Female	African American	dcSSc	0.2	12	1
Patient 3	44	Female	Caucasian	dcSSc	2.3	22	2
Patient 4	54	Male	Caucasian	dcSSc	5.6	11	2
Patient 5	56	Female	Caucasian	dcSSc	2.4	11	0
Patient 6	25	Female	Caucasian	dcSSc	4.1	25	2
Patient 7	51	Male	Hispanic	dcSSc	3.4	16	2
Patient 8	51	Female	Caucasian	dcSSc	4.3	3	0
Control 1	55	Female	Caucasian	Control	N/A	N/A	N/A
Control 2	49	Male	Caucasian	Control	N/A	N/A	N/A
Control 3	50	Female	Caucasian	Control	N/A	N/A	N/A
Control 4	36	Female	African American	Control	N/A	N/A	N/A

Age, disease duration, mRSS, and local skin score were recorded at the time of biopsy. Local skin scores were taken adjacent to the site of the biopsy.

Supplementary Table 5. Average *SIX1* expression extracted from scRNA-seq data from Tabib et al., Nat Commun 2021

	Control (n=10)	SSc (n=12)	Difference (SSc - Control)	Percent Change (SSc vs Control)
MYOC/C7 Fibroblast	0.068	0.165	0.097	142%
STEAP4 Pericyte	0.018	0.070	0.051	288%
CCL19/C7 Fibroblast	0.010	0.024	0.015	149%
COL11A1 Fibroblast	0.003	0.016	0.013	413%
SFRP2 Fibroblast	0.008	0.018	0.010	130%
RERGL Pericyte	0.031	0.041	0.010	30%
Proliferating cells	0	0.009	0.010	
CRABP1ASPN Fibroblast	0.005	0.009	0.003	65%
KRT5 Keratinocyte	0	0.002	0.002	
CA6 Secretory	0	0.002	0.002	
Basal keratinocytes	0	0.001	0.001	
Macrophage/DC	0	0.0007	0.0007	
Terminal Keratinocytes	0.0005	0.0002	-0.0003	-55%
Endothelial cells	0.007	0.005	-0.002	-31%
SCGB1B2P Secretory	0.003	0	-0.003	
SCGB1D2 Epithelial	0.009	0	-0.01	
Preadipocyte	0.010	0	-0.010	
ANGPTL7 Fibroblast	0.019	0	-0.019	

Table 5. Extracted scRNA-seq data from Data4 derived from Tabib et al., Nat Commun 2021. SIX1 expression levels from different cell types identified by cell markers from Tabib et al., Nat Commun 2021. No Difference between SSc and control in the following cell types, T Cell/NK Cell, KRT6A Keratinocyte, Smooth Muscle Cell,

Neural cells, Mast cells, Melanocytes, KRT2 Keratinocyte, Lymphatic Endothelial, RAMP2 Endothelial, Cornified Envelope, Schwann Cell, Plasma cell
