# Supplementary material for: Adipocyte-specific deletion of sine oculis homeobox homolog 1 inhibits lipolysis and reduces skin fibrosis
Source: JCI Insight. 2026 Feb 19;11(7):e181427. doi: 10.1172/jci.insight.181427 (PMC13135399; doi:10.1172/jci.insight.181427)
Supplement: Unedited blot and gel images [file jciinsight-11-181427-s013.pptx]

## Slide 1
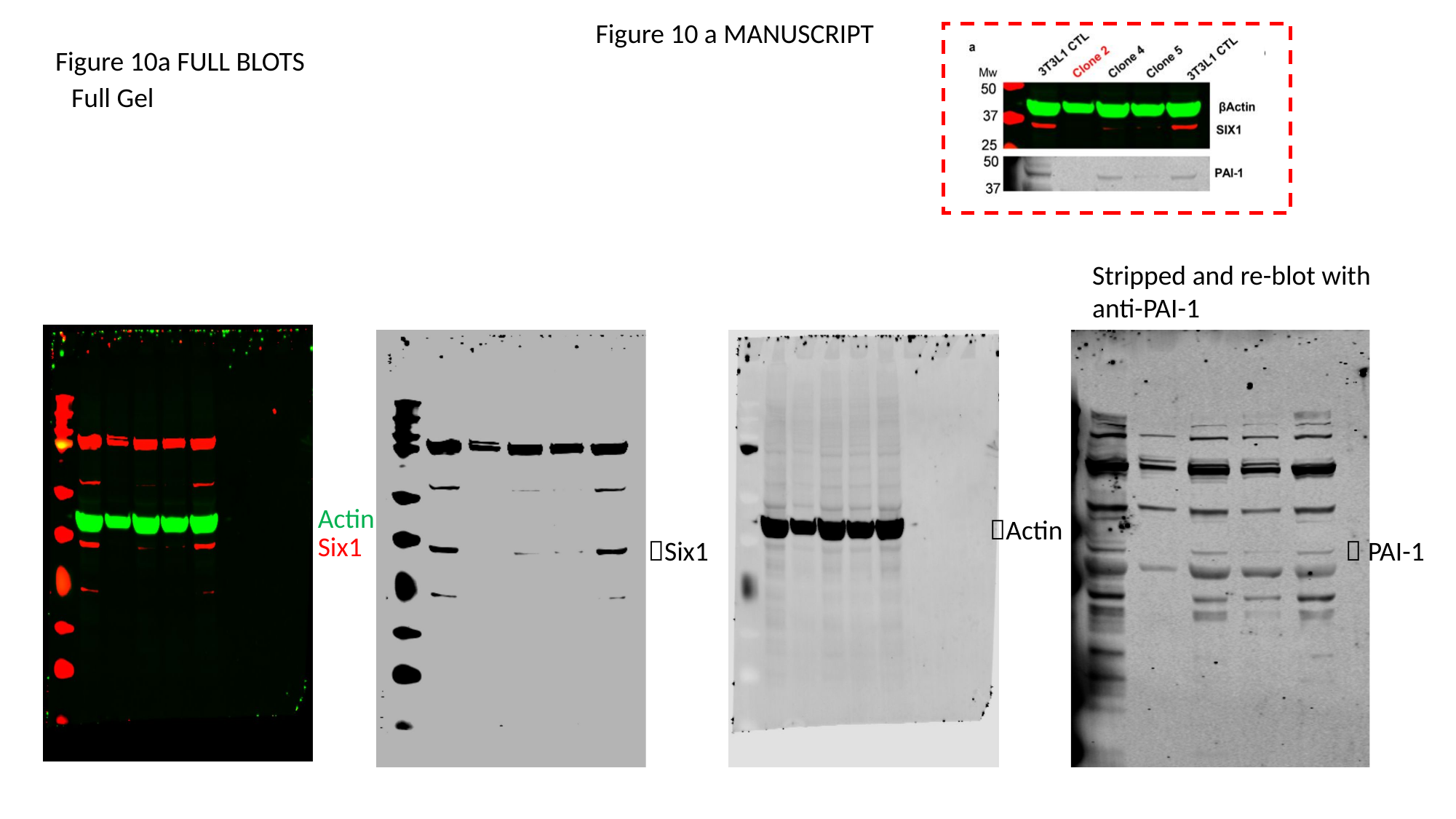

Figure 10 a MANUSCRIPT
Figure 10a FULL BLOTS
Full Gel
Stripped and re-blot with anti-PAI-1
Actin
Actin
Six1
Six1
 PAI-1

## Slide 2
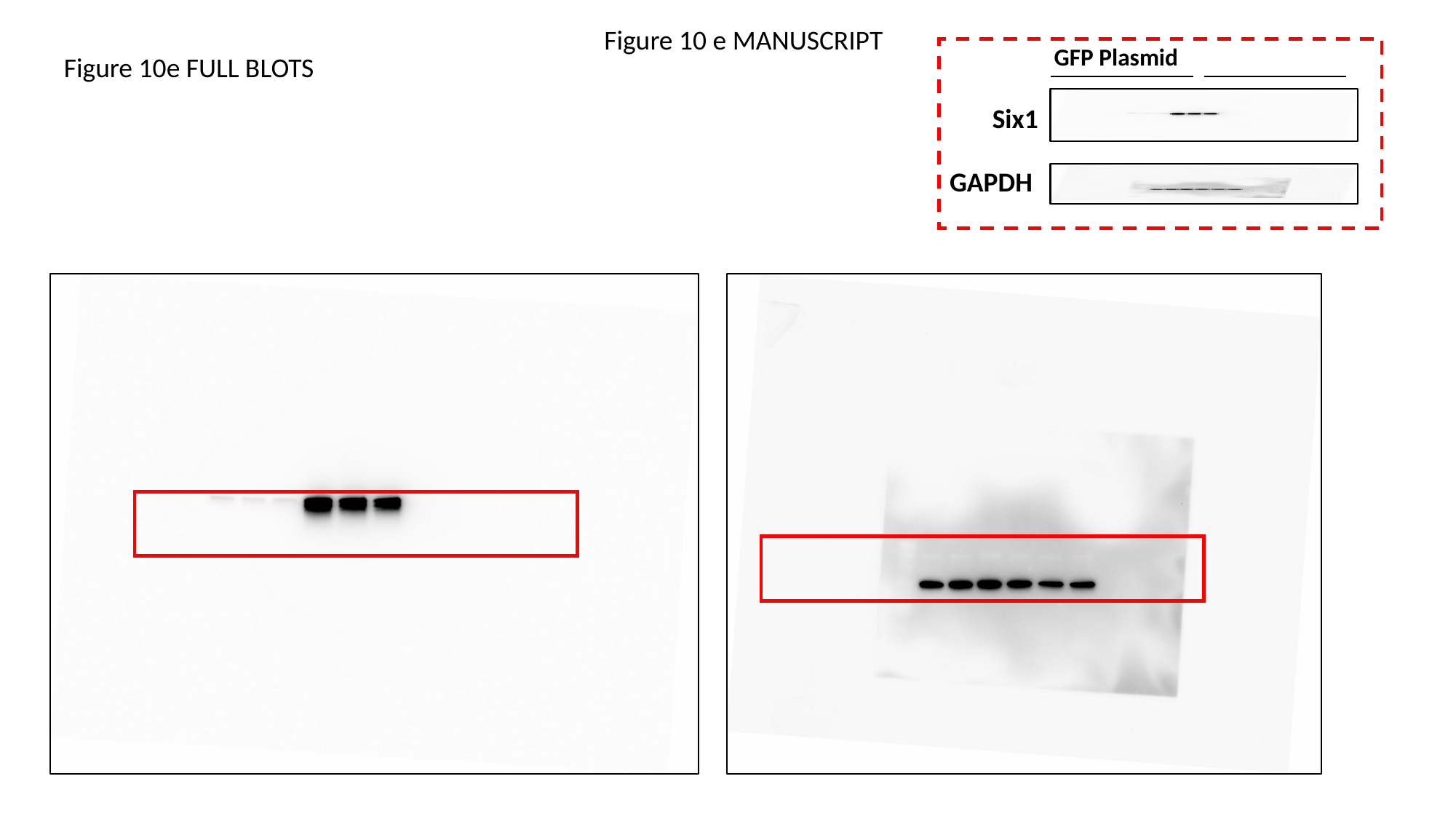

Figure 10 e MANUSCRIPT
GFP Plasmid
Figure 10e FULL BLOTS
Six1
GAPDH

## Slide 3
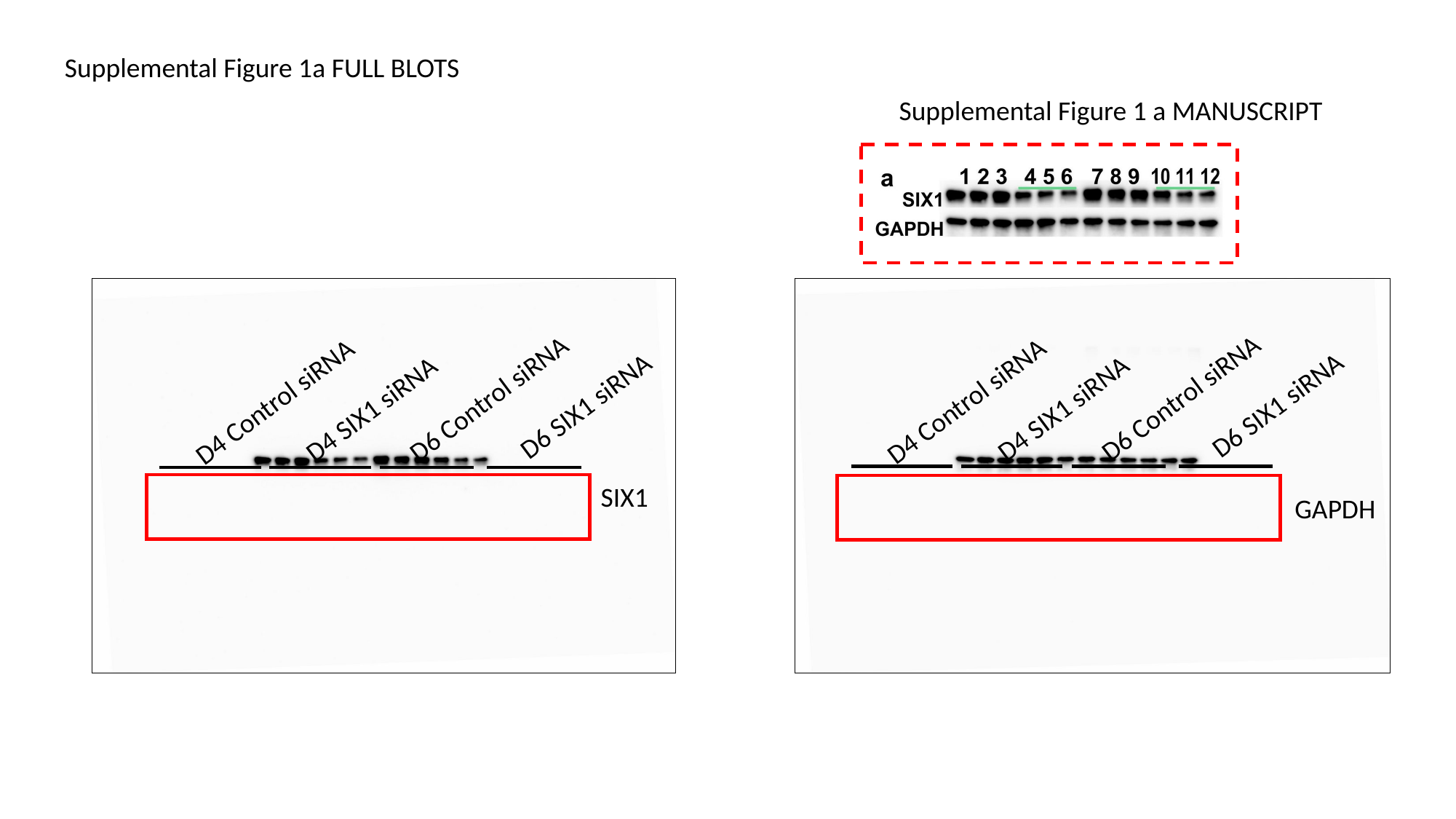

Supplemental Figure 1a FULL BLOTS
Supplemental Figure 1 a MANUSCRIPT
D6 Control siRNA
D6 Control siRNA
D4 Control siRNA
D4 Control siRNA
D6 SIX1 siRNA
D6 SIX1 siRNA
D4 SIX1 siRNA
D4 SIX1 siRNA
SIX1
GAPDH
